# Genome-wide interaction target profiling reveals a novel *Peblr20*-eRNA activation pathway to control stem cell pluripotency

**DOI:** 10.7150/thno.39093

**Published:** 2020-01-01

**Authors:** Cong Wang, Lin Jia, Yichen Wang, Zhonghua Du, Lei Zhou, Xue Wen, Hui Li, Shilin Zhang, Huiling Chen, Naifei Chen, Jingcheng Chen, Yanbo Zhu, Yuanyuan Nie, Ilkay Celic, Sujun Gao, Songling Zhang, Andrew R. Hoffman, Wei Li, Ji-Fan Hu, Jiuwei Cui

**Affiliations:** 1 Key Laboratory of Organ Regeneration and Transplantation of Ministry of Education, Stem Cell and Cancer Center, The First Hospital of Jilin University, Changchun, Jilin 130061, P.R. China.; 2 Stanford University Medical School, VA Palo Alto Health Care System, Palo Alto, CA 94304, USA.; 3 Department of Endocrinology, Xiangya Hospital, Central South University, Changsha, Hunan, P.R. China.

**Keywords:** Stem Cell, pluripotency, enhancer RNA, long noncoding RNA, epigenetics, DNA demethylation

## Abstract

**Background:** Long non-coding RNAs (lncRNAs) constitute an important component of the regulatory apparatus that controls stem cell pluripotency. However, the specific mechanisms utilized by these lncRNAs in the control of pluripotency are not fully characterized.

**Methods:** We utilized a RNA reverse transcription-associated trap sequencing (RAT-seq) approach to profile the mouse genome-wide interaction targets for lncRNAs that are screened by RNA-seq.

**Results:** We identified *Peblr20* (*Pou5F1* enhancer binding lncRNA 20) as a novel lncRNA that is associated with stem cell reprogramming. *Peblr20* was differentially transcribed in fibroblasts compared to induced pluripotent stem cells (iPSCs). Notably, we found that *Peblr20* utilized a *trans* mechanism to interact with the regulatory elements of multiple stemness genes. Using gain- and loss-of-function experiments, we showed that knockdown of *Peblr20* caused iPSCs to exit from pluripotency, while overexpression of *Peblr20* activated endogenous *Pou5F1* expression. We further showed that *Peblr20* promoted pluripotent reprogramming. Mechanistically, we demonstrated that *Peblr20* activated endogenous *Pou5F1* by binding to the *Pou5F1* enhancer in *trans*, recruiting TET2 demethylase and activating the enhancer-transcribed RNAs.

**Conclusions:** Our data reveal a novel epigenetic mechanism by which a lncRNA controls the fate of stem cells by *trans*-regulating the *Pou5F1* enhancer RNA pathway. We demonstrate the potential for leveraging lncRNA biology to enhance the generation of stem cells for regenerative medicine.

## INTRODUCTION

Pluripotent stem cells (PSCs) are vital tools for developmental biology and regenerative medicine due to their unlimited self-renewal capacity and pluripotency 1, 2. PSCs can be isolated as embryonic stem cells (ESCs) from the inner cell mass (ICM) of mammalian blastocysta 3, or somatic cells can be reprogrammed as induced pluripotent stem cells (iPSCs) by exposure to a cocktail of transcription factors (*Pou5F1*, *Sox2*, *Klf-4*, *c-Myc*) 4. The reprogramming process to make iPSCs, however, is extremely inefficient and time-consuming, hindering their potential clinical applications for regenerative medicine. Therefore, it is important to understand the regulatory network that controls their pluripotent state and the reprogramming process.

Long noncoding RNAs (lncRNAs) may play a role in regulating the induction and maintenance of pluripotency 5-8. LncRNAs are a group of >200 ribonucleotide transcripts that do not encode protein products 9. The number of lncRNAs is correlated with the complexity of an organism 10 and lncRNA diversity is restricted to specific cell lineages 11, and it has been postulated that lncRNAs play a role in cell fate determination, including stem cell differentiation and the somatic reprogramming process 7, 8, 12. An understanding of lncRNA physiology could yield novel RNA-based strategies for manipulating ESCs or iPSCs to advance regenerative medicine.

In this communication, we utilized a recently reported RNA reverse transcription-associated capture sequencing (RAT-seq) and RNA-seq strategy to characterize functional lncRNA candidates that are associated with pluripotency 13, 14. By integrating these two approaches, we identified a *Pou5F1* enhancer binding lncRNA, *Peblr20*, that is essential for the maintenance of stem cell pluripotency. We show that *Peblr20* harnesses a novel epigenetic mechanism to control pluripotency in *trans* by recruiting TET2 to *Pou5F1* enhancer loci, thereby activating the enhancer.

## MATERIAL AND METHODS

### Identification of differentially expressed lncRNAs in reprogramming by RNA-seq

As previously reported 13, 14, a combined RNA-seq and RAT-seq strategy was employed to identify pluripotency-associated lncRNAs. The conventional RNA-seq approach was initially used to identify lncRNAs that are differentially expressed during the process of pluripotent reprogramming. Mouse fibroblasts were reprogrammed into iPSCs by using lentiviral *Pou5F1*- Sox2-Klf4-c-Myc-GFP (OSKMN) 15, 16. The selected iPSC colony cells were characterized by immunostaining for stem cell markers, alkaline phosphatase staining, karyotype analysis, and teratoma formation 15, 16.

For RNA-seq, total RNA was isolated from fibroblasts and iPSCs using TRIzol (Invitrogen, Carlsbad, CA), and indexed libraries were prepared using Illumina's TruSeq RNA Sample Prep Kit v2. Paired-end sequencing was performed by Shanghai Biotechnology (Shanghai, PRC) using a HiSeq4000 (Illumina). After Seqtk filtering, clean reads were mapped to the mouse genome (genome version: mm10, GRCm38.p4 (ftp://ftp.ensembl.org/pub/release-83/fasta/mus_musculus/dna/Mus_musculus.GRCm38.dna.primary_assembly.fa.gz) for mRNAs and lncRNAs using the STAR software 17. Gene counts were normalized to the values of Reads Per Kilobase of transcript per Million mapped reads (RPKM). Cuffdiff was used to calculate RNAs that are differentially expressed in reprogramming, using the fold-change > 2 and p < 0.05 with an unpaired two-sided t-test 13.

### Profiling the Peblr20 genome-wide gene targets by RAT-seq

A RAT-seq approach 18, 19 was modified to map the genome wide interacting target genes for lncRNA candidates 13. Briefly, 1.0 × 107 cells were cross-linked with 2% formaldehyde and lysed with cell lysis buffer (10 mM Tris [pH 8.0], 10 mM NaCl, 0.2% NP-40, 1X protease inhibitors). Nuclei were collected, suspended in 1X reverse transcription buffer in the presence of gene-specific primer, biotin-14-dCTP, RNase inhibitor and Maxima Reverse Transcriptase (Thermo Fisher Scientific, CA). After 30 min of reverse transcription of *Peblr20* lncRNA labeled by biotin-14-dCTP with Maxima Reverse Transcriptase at 65◦C, the reaction was stopped by adding 4ul 0.5M EDTA. After nuclear lysis, the complex was subjected to sonication for 180 s (10 s on and 10 s off) on ice with a Branson sonicator with a 2-mm microtip at 40% output control and 90% duty cycle settings. The biotin-cDNA/chromatin DNA complex was pulled down with biotin-streptavidin magic beads (Invitrogen, CA). After reversing the cross-links and washing with 10 mg/ml proteinase K at 65°C overnight and treatment with 0.4 μg/ml RNase A for 30 min at 37°C, the genomic DNA that interacts with the lncRNA was extracted and digested by MboI, and ligated with the NEBNext adaptors (NEBNext® ChIP-Seq Library Prep Master Mix Set for Illumina) to construct the library. The library DNAs were subjected to Illumina sequencing (Shanghai Biotechnology, Shanghai) and PCR with primers shown in **Table S1**. For RAT-seq control, we performed a RAT assay by replacing *Peblr20* complementary primers with random primers and constructed a control library for sequencing using the same protocol.

After RAT sequencing, the low quality reads were filtered using Fastx (version: 0.0.13) software (http://hannonlab.cshl.edu/fastx_toolkit/index.htm). Clean reads were mapped to the mouse genome (genome version: mm10) using the Bowtie (version: 0.12.8) software with default parameters 20. Enriched regions of the genome were identified by comparing the RAT-seq peaks to input samples using MACS2 (version: 2.1.1) and q-value of 0.05 was used as the initial cutoff threshold to minimize peak caller bias 21. The upstream 2 k of the transcription start sites and the downstream 5k of the transcription termination region were defined as the gene regions. The significant GO terms of biological processes with a p-value < 0.05 were selected. We also used the MEME suite 22 for the discovery and analysis of the peaks' sequence motifs. The resulting coverage tracks (bedgraph file) were visualized in UCSC genome browser. To reduce the background, the RAT-seq data were further normalized over the peaks of the control RAT-seq data that were generated by using random oligonucleotide primers in the RAT assay. Differential binding analysis was performed with the DiffBind package using parameters of fold change difference ≥2 and p-value < 0.05, with false discovery rate (FDR) <0.1. The adjusted RAT-seq data were used for mapping the lncRNA target gene interaction network 14.

### RNA extraction and cDNA synthesis

To examine the role of lncRNAs, we collected cells at different stages of reprogramming, including fibroblasts and iPSCs. For comparison, the fibroblast-like cells that expressed OSKMN but failed to complete reprogramming were also collected as the non-iPSCs and used in parallel with iPSCs in the study 23. Total RNA was extracted by Trizol reagent (Invitrogen) according to the manufacturer's guide. Concentration and quality of all RNA samples were evaluated by Nanodrop 1000 (Thermo Scientific,CA), and the 260/280 and 260/230 values of all samples were more than 1.8 and 1.9, respectively. The extracted RNA samples were stored at -80°C. cDNA was synthesized using M-MLV reverse transcriptase (Invitrogen) after genomic DNA digestion. Briefly, 400-800ng total RNA were added to 12ul liquid wax and genomic DNA contamination was removed by DNase I (Millipore Sigma, MA). The reverse transcription reaction was performed with M-MLV reverse transcriptase at 37°C 1h, followed by 95°C 10min. After 10-fold dilution, cDNA was stored at -20°C and ready for PCR.

### Quantitation of gene expression by Q-PCR

Real time PCR was carried out using 3 X Klen-Taq I Mix with a Bio-Rad Thermol Cycler. PCR amplification was performed by PCR of 1 cycle at 95°C for 5 min, 32 cycles at 95°C for 20s, 62°C for15s and 72°C for 15s, and 1 cycle at 72 °C for 10 min. β-Actin was used as PCR input. Quantitative real-time PCR (RT-Q-PCR) was performed using the FastStart Universal SYBR Green Master mix (Millipore Sigma, MA) with a StepOnePlus real-time PCR system (ABI Prism 7900HT; Applied Biosystems, USA). For quantitative real-time PCR, the threshold cycle (Ct) values of target genes were normalized over the Ct of the β-Actin control. Primers used for real-time PCR and Q-PCR are listed in **Table S1**.

### Preparation of cytoplasmic and nuclear fractions

iPSCs were briefly treated with Trypsin-EDTA and gently resuspended in DMEM. Cells were spun down and washed with PBS. After completely aspirating the PBS, 800 μL hypotonic buffer (10 mM Hepes, pH 7.9, 1.5 mM MgCl_2_, 10 mM KCl) were added and placed in ice for 2 min. 10% Nonidet P-40 was added to a final concentration of 0.4% (35 μL). Samples were inverted a few times and spun at 3,000 rpm for 7 min. Supernatants (cytoplasmic fractions) were collected for processing, and the pellet (nuclear fraction) was gently resuspended in 500 μL hypotonic buffer and spun at 3,000 rpm for 2 min. This washing step was repeated three to four times. Both the cytoplasmic and nuclear fractions were processed for RNA extraction, cDNA synthesis and real-time PCR. To verify that the cytoplasmic and nuclear fractions were completely separated, we used U6 as a nuclear control and β-Actin as cytoplasmic control. The primers for PCR are listed in **Table S1**.

### 5'- and 3'-RACE of Peblr20 lncRNA

The full length of *Peblr20* lncRNA was characterized by 3'- and 5'-. The 3'-end was raced using the 3'-race system for rapid amplification of cDNA ends kit (Invitrogen) according to instruction manual. 1^st^ PCR and 2^nd^ PCR were performed by 3' gene-specific primer (GSP) and 3' nested GSP, respectively. The 5'-end was raced using RAT library sample which contained *Peblr20* cDNA. Universal primer from Library Prep Master Mix Set was used as forward primer and 5' GSP and 5' nest GSP were reverse primers for 1^st^ and 2^nd^ PCR respectively. The 5′ - and 3′ -RACE 2^nd^ PCR products were cloned into pJet vector (Thermo Fisher Scientific, CA) and sequenced. All these primers are shown in **Table S1**.

### Lentiviral overexpression of Peblr20 lncRNA in fibroblasts

Using the 3' and 5' race results, full-length 746bp *Peblr20* lncRNA was amplified with PCR primers containing the EcoRI and EcoRV restriction sites. The PCR products were gel-purified, cut by restriction enzymes and ligated into the pCMV-DsRed/Puro vector constructed in our lab. The *Peblr20* lncRNA clone was confirmed by sequencing and then packaged in 293T packing cells 24 using the method described in our lab 25. After transfecting fibroblasts, cells were selected by 1ug/µl puromycin. The DsRed reporter in the vector was used to track the lentivirus transfection efficiency. After 10-day selection, cells were collected for RNA quantitation of *Peblr20* lncRNA and targeted genes using RT-Q-PCR and for DNA methylation.

### Knockdown of Peblr20 lncRNA in iPSCs

*Peblr20* lncRNA was knocked down by shRNA lentiviruses. The shRNA vector was constructed by cloning four shRNAs into pGreenPuro vector (#SI505A-1, SBI, CA). shRNAs were designed online (http://katahdin.cshl.edu/homepage/siRNA/RNAi). For cloning, two pairs of shRNAs (5'- GCCGTTGAGAGTTCAAAGGAAGTTG-3'; 5'-CAACTTCCTTTGAACTCTCAACGGC-3' and 5'-CTGGCTTGCTTTGCTTTGCTAAATA-3'; 5'-TATTTAGCAAAGCAAAGCAAGCCAG-3') combined with loop were linked to the H1 and U6 promoter using PCR and were ligated into the EcoR1/BamH1 site in pGreenPuro vector. The copGFP reporter in the vector was used to track the lentivirus transfection in iPSCs. A random shRNA (GCAGCAACTGGACACGTGATCTTAA) was cloned in the same vector as the assay control (shCT). After lentiviral transfection, iPSCs were selected by puromycin. Nanog was stained to show pluripotent differences between *Peblr20* down-regulated iPSCs and normal iPSCs. Single colonies (SCs) were selected and cultured for expansion. Cells were collected for RNA quantitation of *Peblr20* lncRNA and targeted genes using RT-Q-PCR.

### Immunohistochemical staining of stem cell markers

Immunofluorescent staining was used to examine stem cell markers in iPSC colonies^15^. Cells were briefly rinsed by freshly prepared PBS, fixed by freshly made 4% paraformaldehyde for 10min at RT, washed three times in PBS for 5 min each, permeabilized with freshly made 0.5% v/v Triton X-100/PBS on ice for 5min, then blocked in 1% w/v BSA for 30min at RT. After incubation with primary antibodies diluted in 1% BSA (2ug/ml 1:500) for 1-3h at RT, the samples were washed three times in PBS for 5 min each, incubated with secondary antibody for 1h at RT. The following antibodies were used in the immunostaining: rabbit anti-NANOG (1:100 dilution, Santa Cruz). The cell samples were subsequently incubated with Cy3 or Alexa Fluor 488 labeled secondary antibodies for 1 hour. After washing three times with PBS, samples were counterstained with Hoechst 33258 (Invitrogen). Alternatively, the pluripotency of stem cells was examined by Fluorescent Mouse ES/iPS Cell Characterization kit (Cat.#SCR077, Millipore, MA) following the protocol provided by the manufacturer. Fluorescence images were acquired with a Zeiss AxioCam Camera.

### Embryoid body differentiation

E14 cells were cultured by the hanging drop method in a 10cm culture dish without LIF. After 3 days, embryoid bodies (EBs) were transferred to 10cm ultra-low-adherence plates for suspension culture with slow shaking for up to 8 days. The media were replenished by sedimentation every other day. EBs were collected on D0, D2, D4, D6 and D8 for quantitation of *Peblr20* lncRNA and targeted genes using RT-Q-PCR.

### The Pou5F1 promoter-luciferase assay

The function of *Peblr20* in activating the *Pou5F1* enhancer was first examined in 293T cells by using a dual-luciferase reporter assay. A 3.9 kb genomic DNA fragment covering the *Pou5F1* enhancer, promoter and part of the exon 1 sequence was amplified by PCR using primers: SJ559 5'-TATCGATAGGTACCGTCTGTGAGGAGGTGGCTGAACT-3' and SJ560 5'-ATCGCAGATCTCGAGCTCCTCGGGAGTTGGTTCCAC-3'. The DNA fragment was cloned into pGL3 vector by Kpn1/Xho1.

For the luciferase assay, cells were seeded at a density of 5 × 10^4^ cells/well in 96-well plates. The lentiviral *Peblr20* overexpression vector was co-transfected with a *Pou5F1*-luciferase plasmid and Renilla luciferase control plasmid (Promega) using Lipofectamine 3000 (Invitrogen, CA). The empty lentiviral vector and random lncRNA vector were used as controls. Forty-eight hours after transfection, firefly and Renilla luciferase activities were measured with the dual- luciferase reporter system (Promega) using a luminometer (Turner Biosytem, CA). The relative activity of the *Pou5F1* enhancer was calculated by setting the untreated control cells as 1. All luciferase assays were repeated three times with three culture replicates each.

### The DOX-OSKM reprogramming assay

*Peblr20* cDNA was cloned into pCMV-DsRed-Puro vector and lentiviruses were packaged in 293 cells. Control lentiviruses carried the pCMV-DsRed-Puro empty vector (Vector). OG2 MEFs were transfected with the *Peblr20* and control lentiviruses and were selected by puromycin. MEFs were reprogrammed following the method as described 26. Briefly, 15,000 lentivirus-transfected MEFs were seeded in 12-well plates and were cultured in KSR iPS medium containing 2 μg/ml doxycycline (DOX). The medium was changed every other day. The iPSC colonies were immunostained with rabbit anti-NANOG Antibody (A300-397A, Bethyl, 1:500 dilution). Positive iPSC colonies per field were recorded^15^.

### Status of CpG DNA methylation by sodium bisulfite sequencing

Genomic DNA was extracted by solution D from normal fibroblasts, fibroblasts transfected with vector control and fibroblasts that overexpressed *Peblr20*. DNA bisulfite (BS) conversion was performed using the EZ DNA Methylation-Gold kit (Zymo Research, Orange, CA) according to manufacturer's protocol. PCR was performed using BS-treated DNA samples as template, and BS-specific primers were shown in **Table S1**. PCR conditions were 95°C for 10 min followed by 38 cycles of 95°C for 20s, 58°C for 20s of annealing, 72°C for 20s of extension and completing the reaction at 72°C for 5 min. PCR products were run on 2% agarose gels to verify product size, and extracted using QIAquick gel extraction kit (QIAGEN). Purified PCR products were cloned into pJet vector (Thermo Fisher Scientific, CA) and sequenced. PCR products from CpG3 were digested by restriction enzymes HpyCH4*IV* (NEB, MA) and run on 2% agarose gels. The gray values of gel bands were calculated using ImageJ.

### Chromatin immunoprecipitation and ChIP Q-PCR assay

Chromatin immunoprecipitation (ChIP) assays were performed using ChIP Assay Kit (Millipore Sigma, MA) according to the manufacturer's protocol. Briefly, *Peblr20* overexpressed fibroblasts and vector control transfected fibroblasts were collected respectively and fixed for 10 min at 37 °C with 1% formaldehyde, followed in sequence with SDS lysis and DNA shearing, protein and DNA immuneprecipitation, cross-linked DNA reversal and DNA purification, and finally the immunoprecipitated DNA fragments were detected by real-time Q-PCR. Anti-TET1 (Invitrogen, USA) and anti-TET2 antibodies (Abcam, MA) were immunoprecipitated with sheared DNA from *Peblr20* overexpressed fibroblasts and vector control transfected fibroblasts, respectively. Normal mouse IgG (Millipore Sigma, MA) was used as the negative control. The primers for *Pou5F1* enhancer loci are listed in Supplementary **Table S1**. CHIP-Q-PCR was performed with three replicates.

### RNA binding protein immunoprecipitation (RIP) assay

A lncRNA-affinity binding precipitation assay (RIP) 27 was performed to examine the binding of TET2 protein with *Peblr20* lncRNA. RIP was performed using the Magna RIP^TM^ RNA-Binding Protein Immunoprecipitation Kit (Millipore, Germany) according to the manufacturer's instructions. Generally, *Peblr20* overexpressed fibroblasts were collected and lysed using RIP lysis buffer. Then 100ul cell extract was incubated with RIP buffer containing magnetic beads conjugated with anti-TET2 antibody (Abcam,MA). Mouse IgG was used as the negative control and anti-SNRNP70 was as the positive control. The samples were incubated with proteinase K to digest protein and then the immunoprecipitated RNA was isolated. The purified RNAs were detected by reverse transcription Q-PCR. The primers for Q-PCR are listed in in Supplementary **Table S1**. The CHIP-Q-PCR was performed with three replicates. The Ct values were normalized over the input and compared with IgG control.

### Statistical analysis

All experiments were performed in triplicate. The data were expressed as mean ± standard error of mean (SEM) and were analyzed using SPSS software (version16.0, Inc.,IL). The data were analyzed with Student's *t*-test or by one-way analysis of variance, and statistically significant differences by Student's t test.

## RESULTS

### Identification of Peblr20 as a pluripotent lncRNA by combining RAT-seq and RNA-seq

**Figure 1:**
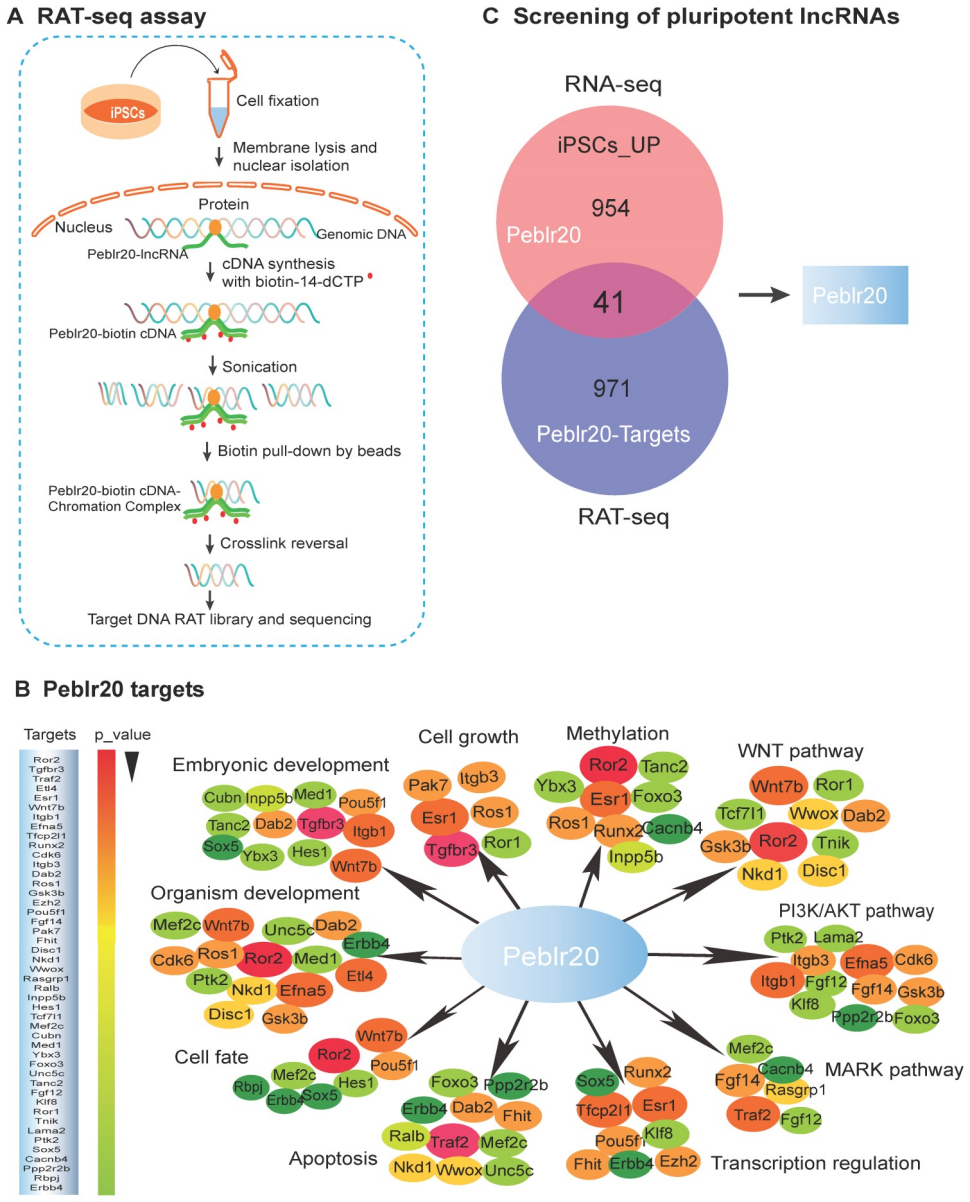
** Identification of pluripotent lncRNAs by RNA-seq and RAT-seq. (A)** Schematic diagram of the RNA reverse transcription-associated trap sequencing (RAT-seq) assay. RNA was *in situ* reverse transcribed using *Peblr20* lncRNA-specific antisense oligo primers and biotin-14-dCTP. The biotin- cDNA/chromatin DNA complex was pulled down by biotin-streptavidin magic bead after sonication. The *Peblr20*-binding chromatin DNAs were isolated for library sequencing. **(B)** The *Peblr20* interaction network targets identified by RAT-seq. *Peblr20* binds to many pathway genes that are involved in embryonic development, transcription regulation, cell fate, signaling pathways, etc. Target genes were listed in the order of *p*-value. **(C)** Profiling pluripotent lncRNAs by integrating RNA-seq and RAT-seq. The conventional RNA-seq approach identifies thousands of lncRNAs that are differentially expressed during pluripotent reprogramming. A modified RAT-seq approach was performed to map the genome wide interacting target genes for the selected lncRNA. Using this strategy, we identified *Peblr20* as a pluripotent lncRNA, because it is activated in iPSCs and binds to multiple stemness gene targets.

LncRNAs play important roles in the growth and self-renewal of stem cells, but their underlying mechanisms of action remain largely uncharacterized. We propose a novel strategy to identify pluripotency-associated lncRNAs (**Fig. S1A**). Conventional RNA-seq is used to identify differentially-expressed lncRNAs related to reprogramming. A RAT-seq assay is then employed to map the genome-wide target interactome with which the lncRNAs interact (**Fig. 1A-1B**). Together, they can identify functional lncRNA candidates that are not only expressed differentially in reprogramming, but are also able to bind to the regulatory elements of multiple core stem cell factor genes (**Fig. 1C, Fig. S1A**) 13, 14.

Using this strategy, we identified a *Pou5F1* enhancer binding lncRNA that matches to 1700097N02Rik. We refer it to as *Peblr20* (*Pou5F1* enhancer binding lncRNA 20). 5'-RACE and 3'-RACE suggested the presence of two variants. *Peblr20* variant 1 shares the same sequences as the four-exon 1700097N02Rik, and variant 2 has two exons, with the 5-end differing from 1700097N02Rik (**Fig. S2**).

RAT-seq and RNA-seq data showed that *Peblr20* is a pluripotency-associated lncRNA. It was silenced in fibroblasts and upregulated in iPSCs in parallel with the stem marker genes, *Pou5F1* and *Sox2* (**Fig. S3**). Notably, *Peblr20* bound to the regulatory elements of multiple stemness genes (**Fig. 1B**), including the *Pou5F1* enhancer (**Fig. S1C**), the *Sox2* enhancer (**Fig. S1D**), and Klf4 CpG islands (**Fig. S1E**). These data suggest that *Peblr20* might play important roles in pluripotency initiation and maintenance by regulating these core factor genes in *trans*.

### Peblr20 lncRNA is required for the maintenance of the pluripotent state

**Figure 2:**
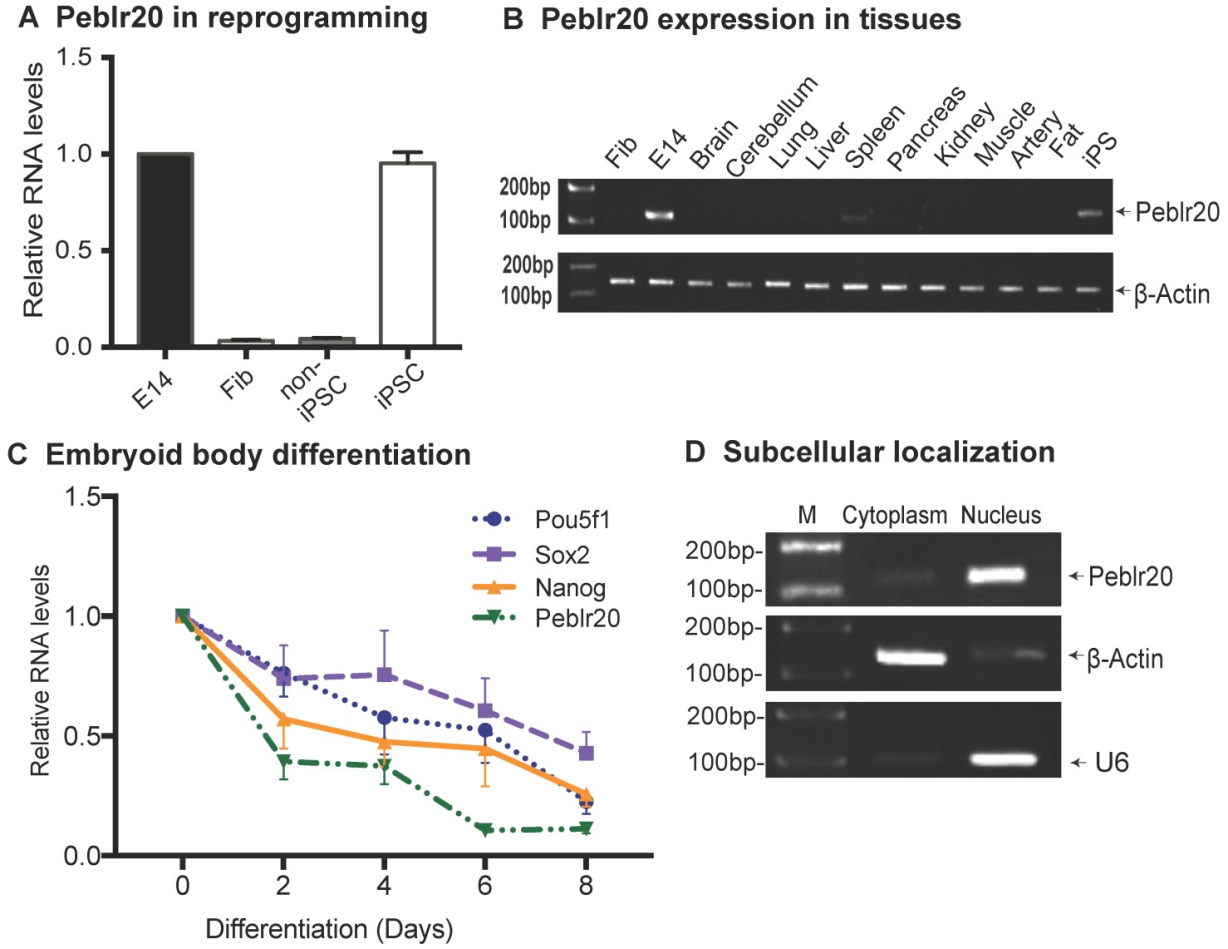
** Characterization of *Peblr20*. (A)** Differential expression of *Peblr20* in reprogramming. Cells were collected at different stages of reprogramming and the dynamic expression of *Peblr20* was measured by RT-Q-PCR. Fib: fibroblast; non-iPSC: un-reprogrammed cells that express four OSKM cocktail factors, but failed to complete reprogramming; iPSC: induced pluripotent stem cells; E14: mouse pluripotent stem cell line used as a positive control. M: marker. Gene expression was normalized to β-Actin. For comparison, positive control E14 was set as 1. **(B)** *Peblr20* lncRNA in differentiated tissues. Ten mouse tissues were collected and cDNAs were synthesized and used for RT-PCR. Fib was used as a negative control, E14 and iPSC were positive controls and β-Actin was used as the internal control. Note that *Peblr20* was not expressed in terminally differentiated tissues. **(C)** Embryoid body differentiation. E14s were collected at D0, D2, D4, D6 and D8 of EB formation for Q-PCR. *Peblr20* was dynamically associated with core pluripotent factors, including *Pou5F1*, *Sox2* and *Nanog*. **(D)** Subcellular localization of *Peblr20* lncRNA. Cytoplasmic and nuclear RNAs were isolated and used to quantitate the subcellular distribution of *Peblr20*. β-Actin was used as the cytoplasmic control and U6 was used as the nuclear control.

In order to verify the role of *Peblr20* in stem cell maintenance, we first examined the dynamic expression of *Peblr20* during pluripotent reprogramming. The expression of *Peblr20* was closely correlated with the pluripotency status during reprogramming (**Fig. 2A**). *Peblr20* was only minimally transcribed in fibroblasts and in non-iPSCs, which carry the OSKM transgenes but fail to complete reprogramming 23. However, *Peblr20* became activated as the cells were fully reprogrammed into iPSCs. Like other pluripotency-associated lncRNAs, *Peblr20* was not expressed in terminally differentiated organs and tissues (**Fig. 2B**).

We also examined the expression of *Peblr20* during the process of embryonic body differentiation, and found that *Peblr20* became down-regulated in parallel with the decreased transcription of the core stem cell factors, *Pou5F1, Sox2* and* Nanog* (**Fig. 2C**). In iPSCs, the cytoplasmic-nuclear fractionation assay revealed that *Peblr20* was predominantly located in the cell nucleus (**Fig. 2D**).

**Figure 3:**
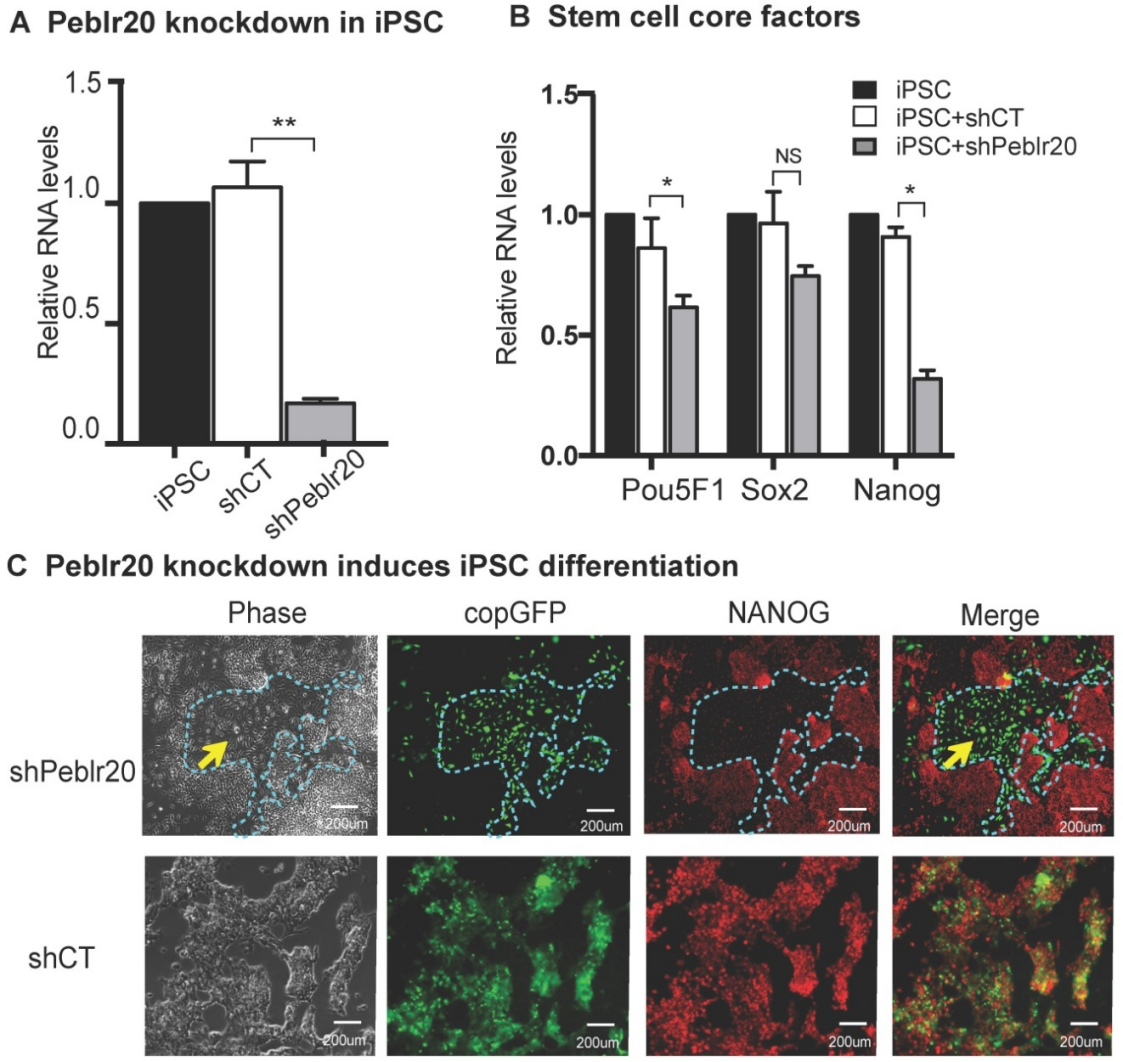
***Peblr20* lncRNA is required for pluripotent state maintenance. (A)** *Peblr20* knockdown in iPSCs. After lentiviral shRNA transfection and puromycin selection, iPSCs colonies were selected, expanded and collected for Q-PCR. shCT: random shRNA control; sh*Peblr20*: *Peblr20* shRNA. For comparison, the abundance of *Peblr20* in iPSCs was set as 1. ** p<0.01 as compared with iPSCs and random shCT controls. **(B)** *Peblr20* knockdown downregulates stem cell core factors in iPSCs. * p<0.05 as compared with iPSC and random shCT controls. NS: not significant. **(C)** *Peblr20* knockdown induces iPSC differentiation. The Obelr20 shRNA and random shRNA lentiviral vectors carry the copGFP reporter gene (green). After lentiviral transfection, cells were fixed and immunostained by an antibody against the stem cell pluripotent marker NANOG (red). Compared to the control group, sh*Peblr20* transfected iPSCs were negative for NANOG immunostaining and were differentiated morphologically (blue dotted line).

We then performed a loss-of function experiment to study the role of *Peblr20* in pluripotent stem cells. Two *Peblr20* shRNAs were constructed under the control of the H1 and U6 promoters, respectively, in a lentiviral vector (pCMV-copGFP/Puro) (**Fig. S4A**). After lentiviral transfection, iPSCs were selected by puromycin. Single colonies emitting the copGFP green signal were selected, expanded and collected for RT- Q-PCR (**Fig. S4B**). *Peblr20* shRNA knockdown reduced *Peblr20* expression by 80% (**Fig. 3A**). After *Peblr20* knockdown, the expression of *Pou5F1, Sox2,* and *Nanog,* were also down-regulated (**Fig. 3B**). The sh*Peblr20*-transfected iPSCs exited from pluripotency, as demonstrated by their differentiated morphology and the loss of immunostaining staining for the pluripotent marker protein NANOG (**Fig. 3C**, top panel, yellow arrow). As the control, the treatment with random shRNA (shCT) did not affect the pluripotency of iPSCs (**Fig. 3C**, bottom panel). These data suggest that *Peblr20* is a crucial molecular factor required for maintenance of pluripotency.

### Peblr20 enhances iPSC reprogramming efficiency

**Figure 4:**
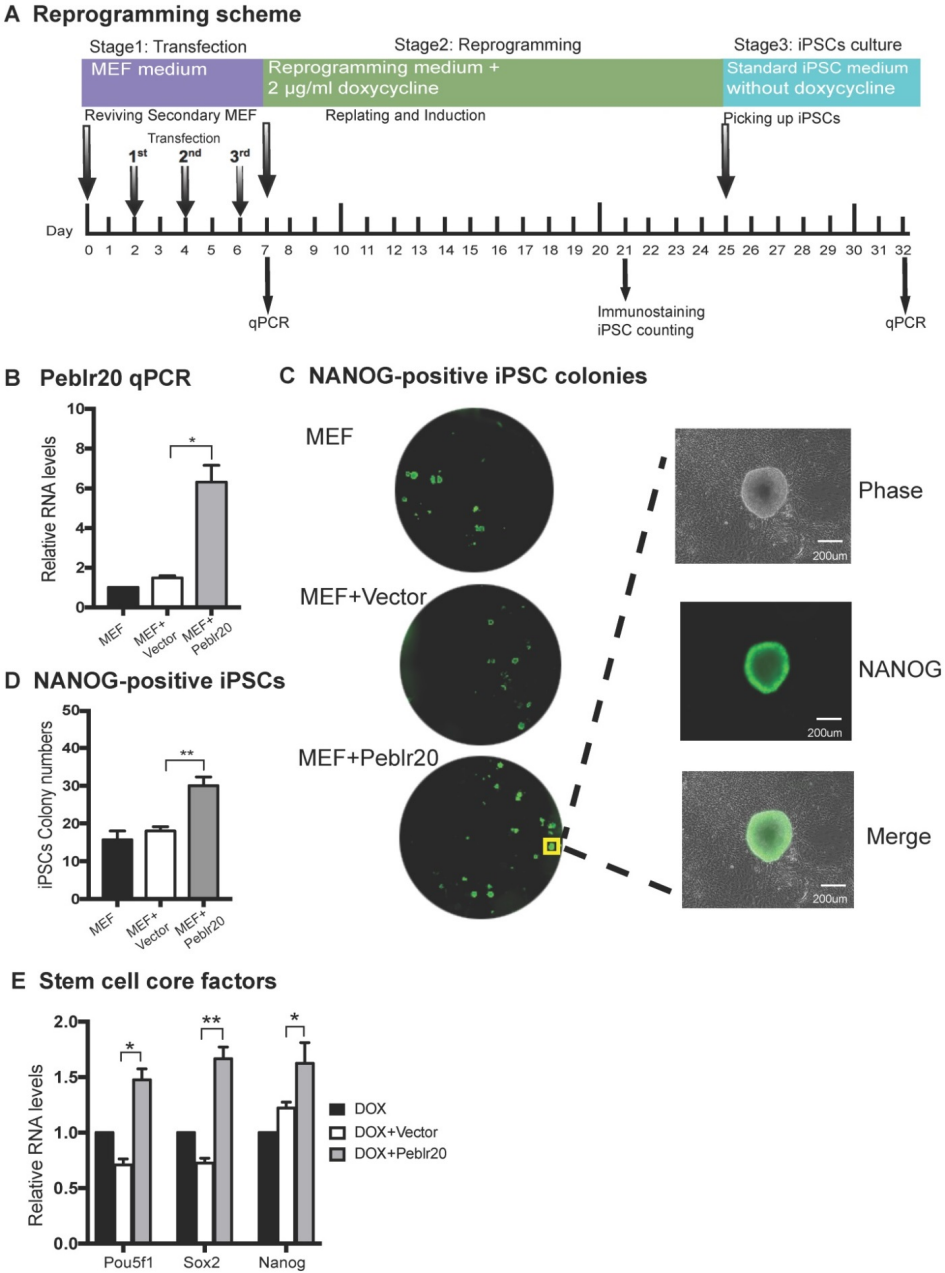
***Peblr20* improves reprogramming efficiency. (A)** Reprogramming scheme. Cells were collected at three separate stages for Q-PCR. After reprogramming, colonies were stained by anti-NANOG. **(B)** Overexpression of *Peblr20*. After transfection, MEFs were collected and used for quantitation of *Peblr20* lncRNA. **(C)** NANOG-positive colonies after immunostaining. Cells from D21 colonies were fixed and stained with NANOG antibody. NANOG-positive colonies/well (left, under 4x objective lens) A single NANOG-positive colony is shown in the right panel (20x objective lens). **(D)** NANOG-positive colonies per well. **(E)** Upregulation of core pluripotent factors in *Peblr20* transfected secondary MEF cells. * P < 0.05; ** P < 0.01.

To determine whether high levels of *Peblr20* transcripts affect reprogramming, we performed a reprogramming assay using secondary mouse embryonic fibroblasts (MEFs) that carry the DOX-inducible lentiviral OSKM 28. MEFs were administered three doses of lentivirus carrying the pCMV-DsRed/Puro-*Peblr20* cassette (**Fig. 4A**). An empty lentiviral vector was used as the control. After puromycin selection, cells were collected to assess the expression of *Peblr20* by RT-Q-PCR (**Fig. 4B**). After it was determined that the abundance of *Peblr20* was increased, we incubated the cells in a medium containing 2µg/ml doxycycline (DOX) as a reprogramming inducer. In MEF cells transfected with the lentivirus containing the *Peblr20* cassette, typical iPSC colonies began to appear on day 15 (eight days after DOX addition). On day 21, reprogramming efficiencies were examined by immunostaining for the stem cell marker NANOG (green fluorescence) (**Fig. 4C**). The number of NANONG-positive colonies was significantly increased in the *Peblr20*-overexpressing group (**Fig. 4D**).

At the conclusion of reprogramming, iPSCs colonies were selected and cultured on mitomycin C-inactivated MEF feeder cells using standard iPSC medium without DOX. iPSCs were collected after the third passage on day 32 for RNA quantitation of *Pou5F1*, *Sox2* and* Nanog*. *Peblr20*-overexpressing cells contained significantly higher abundance of stemness genes (**Fig. 4E**). Taken together, these results indicate that *Peblr20* lncRNA improves reprogramming efficiency.

### Peblr20 activates stem cell core factors in trans

**Figure 5:**
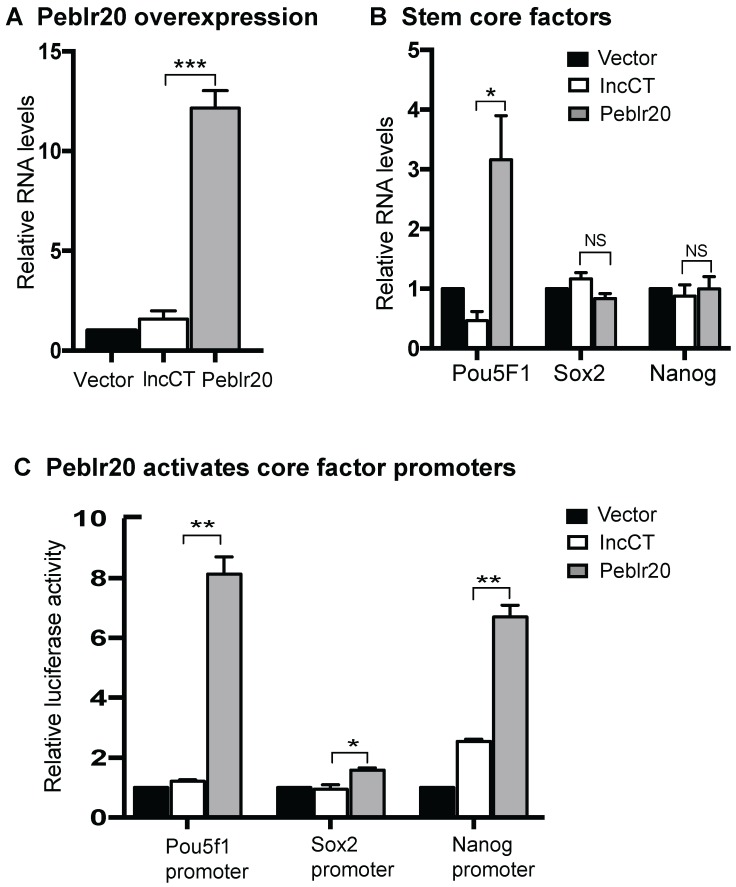
***Peblr20* activates stem cell core factors. (A)** *Peblr20* overexpression in fibroblasts. (B) The effect of *Peblr20* on stem cell core factor genes. (C) *Peblr20* activates promoter activities of core pluripotent factors. 293T cells were co-transfected by reporter plasmids and *Peblr20* plasmid. Forty-eight hours after transfection, cells were collected for luciferase activity measurements. Reporter plasmid empty vector and random lncRNA (lncCT) vectors were used as the controls.

We then initiated a series of studies to delineate the molecular mechanisms by which *Peblr20* regulates reprogramming. First, we examined if *Peblr20* was able to activate the expression of core stem cell factor genes in fibroblasts. We synthesized a lenti pCMV-DsRed/Puro-*Peblr20* plasmid (**Fig. S4C**) and packaged the lentivirus in 293T cells. Fibroblasts were transfected with *Peblr20* lentiviruses. After puromycin selection, ~90% of fibroblasts overexpressed *Peblr20* using DsRed as a tracking marker (**Fig. S4D**). Using RT-Q-PCR, we confirmed a >12 fold overexpression of *Peblr20* as compared with the vector control (**Fig. 5A**). *Pou5F1,* but not *Sox2* or* Nanog,* was significantly upregulated in cells in which *Peblr20* was overexpressed (**Fig. 5B**).

Dual-luciferase reporter systems have been widely used to study the activation of specific core regulatory elements in pluripotent stem cells 29-31. We used this system to confirm whether *Peblr20* lncRNA could enhance the promoter activity of *Pou5F1*, *Sox2* and *Nanog*. We constructed promoter reporter plasmids of these three genes and co-transfected them with a *Peblr20* lncRNA overexpression plasmid and Renilla luciferase control plasmid into 293T cells. Forty-eight hours after transfection, cells were harvested and the luciferase activity was measured using the Dual-Luciferase reporter assay system. As compared with the vector control (Vector) and the random lncRNA control (lncCT), *Peblr20* lncRNA significantly enhanced the activities of the* Pou5F1* and *Nanog* promoters (**Fig. 5C**).

### Peblr20 facilitates Pou5F1 enhancer RNA transcription

**Figure 6:**
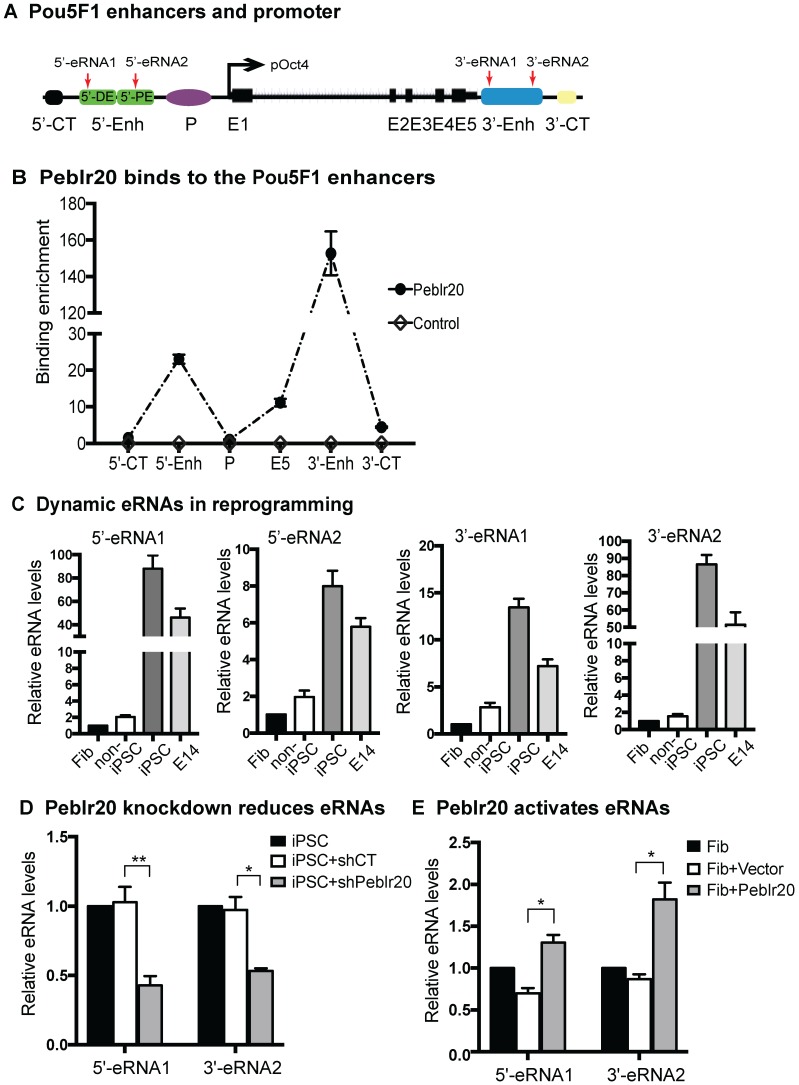
***Peblr20* activates the *Pou5F1* enhancer RNA pathway. (A)** Location of *Pou5F1* enhancer RNAs. 5'-eRNA1: the 5-distal enhancer (5'-DE); 5'-eRNA2: the 5'-proximal enhancer (5'-PE); 3'-eRNA1 and 2: the 3'-enhancer (3'-Enh); P: the *Pou5F1* promoter; 5'-CT, 3'-CT: the RAT 5'- and 3'- control sites; E1-E5: *Pou5F1* exons 1-5. **(B)** *Peblr20* lncRNA-*Pou5F1* DNA interactions by Q-PCR. RAT library samples were used to perform Q-PCR to quantitate binding intensity. The results were normalized to the value of the streptavidin bead pulldown control. Control: the RAT-seq library was constructed using random oligo primers; Obelr20: the RAT-seq library was constructed using *Peblr20*-speccific primers. **(C)** Dynamic expression of *Pou5F1* eRNAs in reprogramming. Cells were collected at various stages of reprogramming and the expression of eRNAs was measured by RT-Q-PCR. Fib: fibroblast; URC: non-iPSC: fibroblasts that carried the lentiviral OSKM but failed to complete reprogramming; iPSC: induced pluripotent stem cells from secondary MEFs; E14: mouse pluripotent stem cell line as a positive control. Gene expression was normalized to β-Actin internal control. **(D)** *Peblr20* knockdown inhibits *Pou5F1* eRNA. iPS+shCT: iPSCs transfected with the random shRNA vector control; iPS +sh*Peblr20*: iPSCs transfected with sh*Peblr20*. Gene expression was normalized to β-Actin control. **(E)** *Peblr20* overexpression activates *Pou5F1* enhancer RNAs in fibroblasts. Cells were collected from gain-of-function experiments and eRNAs expressions were measured by RT-Q-PCR. Fib+Vector: fibroblasts transfected with vector control; Fib+*Peblr20*: fibroblasts overexpressed Peblr20. All experiments were performed in triplicate and statistically significant differences by Student's t test. * P < 0.05; ** < 0.01.

To further delineate the mechanism underlying the activation of *Pou5F1*, we used quantitative PCR to map *Peblr20* binding at the *Pou5F1* locus (**Fig. 6A**). Using RAT-seq, we found that *Peblr20* bound to the *Pou5F1* 5'- and 3'-enhancer elements. No such interaction signals were detected in the RAT random control library products (CTL). As expected, we did not detect the RAT signals at the 5'- and the 3'- control sites (5'-CT, 3'-CT). Interestingly, *Peblr20* did not bind to the *Pou5F1* promoter (p*Pou5F1*) (**Fig. 6B**).

Enhancers are defined as DNA elements that act over a distance to positively regulate expression of target genes in a spatial and temporal fashion 32. Recent studies have shown that active enhancer loci can recruit RNA polymerase II (RNA Pol II) and express enhancer RNAs (eRNAs) and these eRNAs correlate with the enhancer activity. The eRNA-transcribed enhancers are more active than the non-transcribed enhancers in activating their target genes.

To determine if *Peblr20* lncRNA might activate the *Pou5F1* through eRNA transcription, we collected cells during pluripotent reprogramming and used Q-PCR to map the eRNA transcripts at the *Pou5F1* locus, including the 5'- distal enhancer, 5'- proximal enhancer, and 3'- enhancer (**Fig. 6A**). We found that the abundance of eRNA transcripts was closely associated with pluripotency (**Fig. 6C**). The 5'- and 3'- eRNAs, particularly 5'-eRNA1 and 3'-eRNA2, were actively transcribed in iPSCs and embryonic stem cell E14 line, but were expressed at very low levels in fibroblasts and non-iPSCs that expressed the lentiviral OSKM but failed to complete reprogramming 23.

We then knocked down *Peblr20* in iPSCs and performed Q-PCR to measure the *Pou5F1* eRNAs. After *Peblr20* knockdown, we found that the eRNAs at both the *Pou5F1* 5'- and 3'- enhancer sites were significantly downregulated (**Fig. 6D**). The shRNA control (gCT) did not interfere with the eRNA transcription. We also examined whether ectopic expression of *Peblr20* would affect the eRNAs in fibroblasts. As seen in **Figure 6E**, overexpression of *Peblr20* significantly increased the expression of eRNAs at the *Pou5F1* enhancer loci. Taken together, these data suggest that *Peblr20* may regulate the *Pou5F1* gene activity by promoting the eRNA synthesis.

### Peblr20 activates Pou5F1 enhancer RNA expression by inducing DNA demethylation

**Figure 7:**
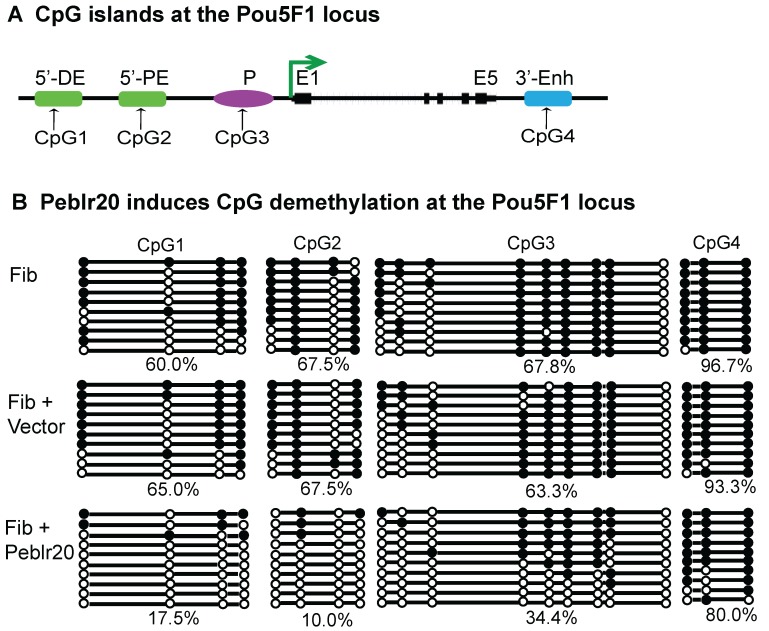
***Peblr20* induces DNA demethylation at the *Pou5F1* enhancer loci. (A)** CpG islands at the* Pou5F1* locus. CpG1: 5'-distal enhancer (5'-DE); CpG2:5' proximal enhancer (5'-PE); CpG3: promoter region (P); CpG4: 3'-enhancer (3'-Enh). **(B)** *Peblr20* induces CpG demethylation at the *Pou5F1* locus. DNAs were extracted from fibroblasts from gain-of-function experiments, treated by sodium bisulfite, amplified with the *Pou5F1*-specific primers, cloned into the pJET vector, and sequenced. Solid circle: methylated CpG; open circle: un-methylated CpG. Each line represents sequencing from one clone. Number under the last line represents the methylation level of each CpG island.

To determine if *Peblr20* induced *Pou5F1* eRNAs by altering epigenetic modifications at the *Pou5F1* enhancer and promoter loci (**Fig. 7A**), we isolated genomic DNAs from fibroblasts that overexpressed *Peblr20* and used sodium bisulfite sequencing to examine the status of DNA methylation. The lentiviral vector control did not affect DNA methylation in these loci, however, after ectopic expression of *Peblr20*, we observed an extensive decrease in DNA methylation (**Fig. 7B**, panel 3). Using a restriction enzyme digestion assay, we also confirmed a 40% reduction of DNA methylation in the *Pou5F1* promoter CpG island in the *Peblr20-*overexpressing fibroblasts (**Fig. S5**). These data suggest that the overexpression of *Peblr20* may trigger the activation of the *Pou5F1* and its enhancer RNAs through DNA demethylation.

### Peblr20 recruits TET2 to the Pou5F1 enhancer locus

The Ten eleven translocation (TET) family of enzymes (TET1/2/3) are critical dioxygenases in DNA demethylation, and they have emerged as potential drivers of epigenetic reprogramming 33, 34. We thus asked if TET family members were involved in DNA demethylation in *Peblr20*-overexpressing fibroblasts. We focused on TET1/TET2, which are correlated with the status of pluripotency in mouse ESCs 35, 36.

**Figure 8:**
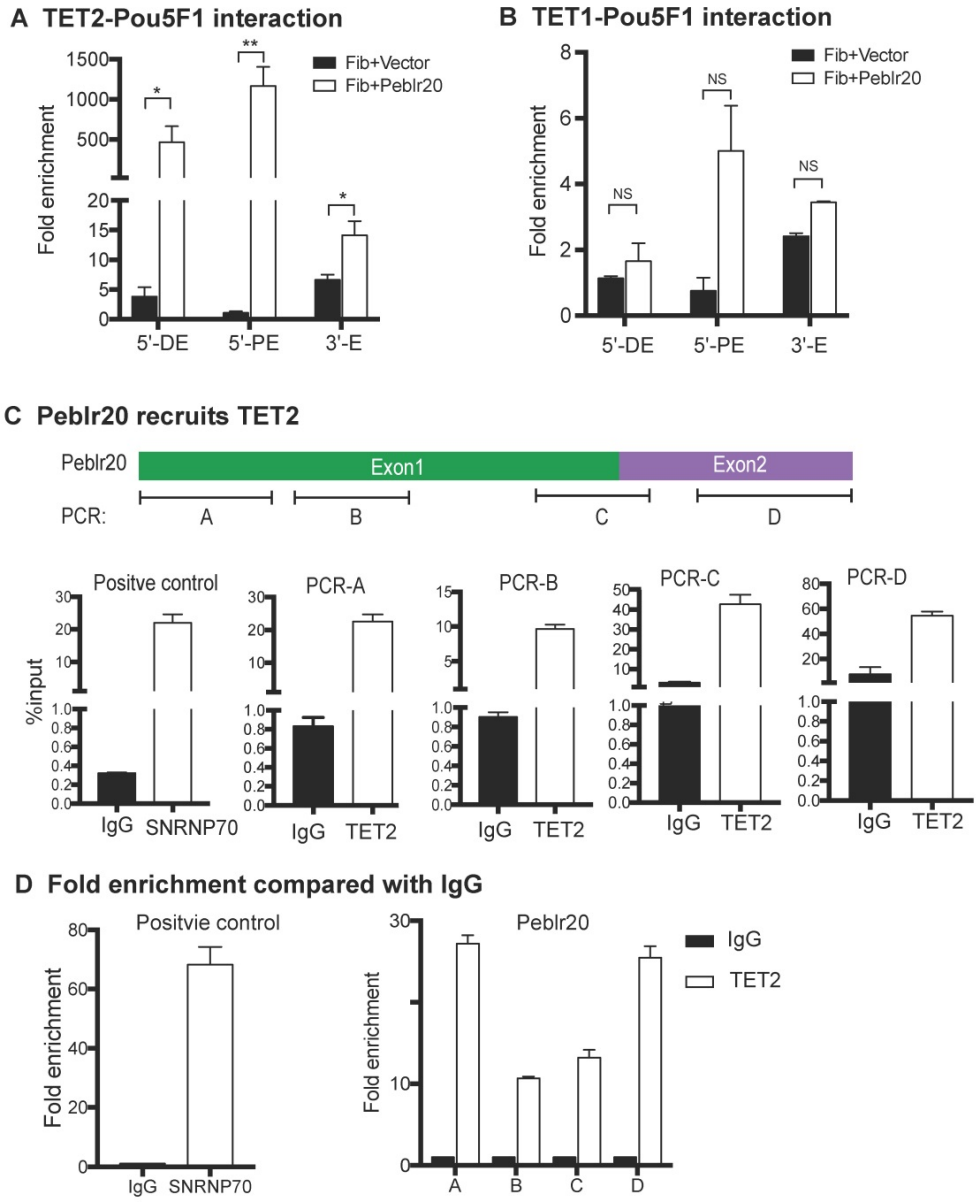
***Peblr20* recruits TET2 to the *Pou5F1* enhancer loci. (A)** Interaction of TET2 at the *Pou5F1* enhancer loci in the *Peblr20* overexpressing fibroblasts. Enrichments of TET2 to the *Pou5F1* enhancers were measured from TET2 ChIP samples and normalized to the IgG control samples. Compared to vector control group, there were remarkable enrichments of *Pou5F1* enhancer loci with TET2 in *Peblr20* overexpressed fibroblasts. 5'-DE: 5'-distal enhancer; 5'-PE: 5'-proximal enhancer; 3'E: 3'-enhancer. **(B)** No binding of TET1 with *Pou5F1* enhancer loci. Fold enrichments of *Pou5F1* enhancer loci were measured using TET1 ChIP samples relative to IgG samples. **(C)** *Peblr20* recruits TET2. Fibroblasts transfected with *Peblr20* were collected and immunoprecipitated with anti-TET2 antibodies, with mouse IgG as negative control and with anti-SNRNP70 as positive control. Purified binding RNAs were reverse transcribed and used as Q-PCR templates. RIP data are represented by fold enrichments relative to the input. **(D)** RIP data represented by fold enrichments relative to IgG showed that TET2 interacted primarily with the 3'- and 5'-fragment of *Peblr20* lncRNA.

First, we examined if TET1/2 interacted with the regulatory elements of the *Pou5F1* gene. Fibroblasts were transfected with lentiviruses carrying *Peblr20* or empty vector control. After puromycin selection, cells were collected and chromatin immunoprecipitation (ChIP) was performed with anti-TET1, anti-TET2 and mouse IgG control antibodies. Real-time PCR was used to measure immunoprecipitation enrichment at the 5'-distal enhancer, 5'-proximal enhancer, and 3'-enhancer of *Pou5F1* after adjusting over the IgG control. TET2, but not TET1, bound to the *Pou5F1* enhancer loci in *Peblr20*-overexpressing fibroblasts (**Fig. 8A-8B**).

We then performed a RNA-binding protein immunoprecipitation (RIP) assay to examine if *Peblr20* interacts with TET2. The TET2-RNA chromatin complex was immunoprecipitated with anti-TET2 antibody. An anti-SNRNP70 antibody was used as the positive control and a mouse IgG was used as the negative control. Immunoprecipitated RNA fragments were reverse transcribed and detected by real-time Q-PCR using primers from *Peblr20* (**Fig. 8 C**). As compared with the IgG control, it seemed that TET2 interacted primarily with the 3'- and 5'-fragment of *Peblr20* lncRNA (**Fig. 8D**). These data suggest that *Peblr20* may help recruit TET2 to the *Pou5F1* locus, where it induces DNA demethylation and promotes eRNA expression.

## DISCUSSION

The specific role of lncRNAs in cell fate determination, particularly the stem cell reprogramming process, remains unclear. Using combined RNA-seq and RAT-seq assays, we have identified *Peblr20* as a critical lncRNA component that helps maintain the pluripotent status of iPSCs. *Peblr20* is differentially transcribed in pluripotent reprograming, being transcriptionally silenced in terminally differentiated fibroblasts but activated in iPSCs. Knockdown of *Peblr20* induces exit from pluripotency in iPSCs. In contrast, overexpression of *Peblr20* activates stemness genes in fibroblasts and enhances pluripotent reprogramming. Most importantly, we demonstrate that *Peblr20* is the first lncRNA that controls the pluripotency of stem cells by epigenetically activating the enhancer RNA pathway.

It is generally believed that lncRNAs may act as decoys, guides and scaffolds to suppress or enhance the function of other regulators, including DNA, RNA and proteins 37, depending on their intracellular localization 38, 39. Nuclear lncRNAs can guide chromatin modification complexes to specific genomic loci and/or serve as molecular scaffolds that tether together distinct functionally related complexes 40. Due to their intrinsic ability to base-pair with other nucleic acids, lncRNAs can exert repressive or promoting activities on target genes by *cis*-acting on neighboring genes or *trans*-acting on genes at distant loci 41. In contrast, some lncRNAs are localized in the cytoplasm, where they can regulate phenotypes through base-pairing complementary regions on RNA targets 42. Depending on their target partners, lncRNAs can regulate chromatin modifications 43, RNA binding proteins 44, miRNA activity 45 and signaling pathways 46. LncRNA binding to target DNA can initiate the formation of heterochromatin by recruitment of DNA or histone methyltransferases, resulting in repression of gene expression 47. Conversely, transcriptional activation can be induced by recruitment of different chromatin modifiers, such as histone H3 lysine 4 (H3K4) and methyltransferase MLL1, or by changing the 3D chromatin conformation 40, 48, 49. Most importantly, we demonstrate in this study that *Peblr20* functions through a unique enhancer RNA regulatory pathway. *Peblr20* specifically interacts with the *Pou5F1* enhancers and facilitates *Pou5F1* eRNA expression. By activating this complicated eRNA regulatory network, *Peblr20* controls the pluripotent state and iPSC reprogramming process.

DNA methylation status at regulatory elements of pluripotency regulator genes, such as *Pou5F1*, is correlated with stable silencing of these genes during development. Overcoming this barrier is believed to be a key step to initiate cellular reprogramming 50-53. Recently, hypomethylation has also been found at active enhancers 54, 55. Using whole-genome bisulfite sequencing (BisSeq), Stadler 55 generated methylomes in mouse ESCs and in neuronal progenitors (NP). Using an analytical approach that quantifies DNA methylation locally, they identified a novel epigenomic feature defined by localized reduced levels of methylation. These low-methylated regions (LMRs) show an average methylation of 30% and occur distal to promoters with little overlap with CpG islands. Contrasting LMRs with maps of histone modifications, DNase I hypersensitivity and several DNA-binding factors revealed that LMRs represent distal regulatory regions, such as enhancers. This is in agreement with several single-gene studies of methylation dynamics at localized distal regulatory regions 56, 57. Most recently, Hon et al 58 identified 302,864 tissue-specific differentially methylated regions (tsDMRs) by profiling the methylomes of a diverse panel of 17 normal adult mouse tissues. The identified tsDMRs are generally hypomethylated in a tissue-specific manner. Most notably, these sequences predominantly correspond with distal regulatory elements in the genome. Moreover, some tsDMRs mark enhancers that were dormant in adult tissues but active in embryonic development. In our study, *Peblr20* promoted DNA demethylation at the *Pou5F1* locus, especially the 5'-enhancer region. After *Peblr20* overexpression, the originally silent *Pou5F1* enhancers became highly demethylated in fibroblasts. Consequently, the *Pou5F1* eRNAs are transcribed, and they activate the *Pou5F1* promoter to promote pluripotent reprogramming.

DNA and chromatin-modifying enzymes establish an ESC/iPSC-specific epigenomic landscape that is linked to a network of pluripotency genes that influences differentiation potential. These enzymes contain an active C-terminal catalytic domain that converts 5-methylcytosine (5mC) sequentially to 5-hydroxymethylcytosine (5hmC), 5-formylcytosine (5fC), and 5-carboxylcytosine (5caC) 59. TET-driven 5mC oxidation provides a direct mechanistic route for both passive and active DNA demethylation. Both Tet1 and Tet2 are expressed in mouse ESCs, while Tet3 is not expressed in ESCs and is only induced upon differentiation, consistent with its presence in various differentiated cell types 35, 36. Tet1 and Tet2 are key players in this network of coordinated genetic and epigenetic control. The generation of Tet1, Tet2, and Tet3 single mutant 60, 61 and Tet1/2 double-knockout (DKO) 35 and Tet1/2/3 triple-knockout (TKO)^62^ mESCs has shed light on the roles of these proteins in pluripotency as well as embryonic and adult development. Depletion of Tet1 or Tet2 significantly reduces iPSC reprogramming efficiency in conventional reprogramming medium 63-65, and triple knockout Tet1/2/3 (Tet-TKO) mouse embryonic fibroblasts completely fail to generate iPSC colonies following OKSM overexpression 65. Whether Tet1 is implicated in promoter and enhancer demethylation and gene activation during iPSC reprogramming has been partially addressed in a study reporting that Tet1 is necessary for the demethylation of the *Pou5F1* enhancer and promoter regions followed by the transcriptional reactivation of this gene 66. In our study, using both ChIP-PCR and RIP-PCR assays, we confirmed that *Peblr20* lncRNA recruits TET2 to the *Pou5F1* enhancers, where DNA demethylation promotes eRNA expression.

It should be noted that not all enhancers are able to transcribe eRNAs. Kim *et al* 67 estimated that only about half of the intergenic enhancers can transcribe eRNAs. Enhancer transcription is a widespread phenomenon observed across multiple cell types in different species 68-71. Using an integrated epigenomic screening, Ounzain and colleagues 72 recently established a catalogue of enhancer RNAs that are dynamically expressed in ESCs during cardiac differentiation. The expression of these transcripts correlated with the expression of target genes in their genomic proximity. The expression of the eRNAs was inhibited when the target mRNA abundance reached maximal levels. Using genome-wide analysis of eRNAs in gene regulation across 12 mouse tissues, Cheng 73 demonstrated that the state of enhancer activity is tissue-specific, as the same enhancer can differentially transcribe eRNAs across tissues. Overall, these data are an important contribution to the functional impact of eRNAs on pluripotency and differentiation. In our study, the transcription of *Pou5F1* eRNAs correlated with the pluripotent state, and transcription changed in parallel with *Peblr20* lncRNA levels.

Recent studies suggest that eRNAs appear to function through three distinct mechanisms. First, eRNAs may facilitate chromosomal looping formation between enhancers and promoters. Chromatin interaction studies demonstrated that enhancers engaged in looping with promoters of protein-coding genes possess higher expression of eRNAs 74. Numerous studies 71, 75, 76 have suggested a potential role of eRNAs in the process of proper formation of chromosomal looping between enhancers and promoters. The potential role of eRNA in the modulation of chromosomal looping was also suggested by the observation that eRNAs could interact with the SMC3 (structural maintenance of chromosomes 3) and RAD21 (double-strand-break repair protein rad21 homolog) subunits of the cohesin complex, which has been shown to control enhancer-promoter looping in stem cells 76. Second, eRNAs play a role in the eviction of transcriptional repressors. A study 77 showed that eRNAs facilitated the transition of paused RNA polymerase II (RNAPII) into active elongation by acting as decoys for the negative elongation factor (NELF) complex upon induction of immediate early genes (IEGs) in neurons. Third, eRNAs recruit transcriptional activators. Mousavi et al 78 found that knockdown of eRNA from core enhancer of *MyoD* locus decreased RNA PolII recruitment at the promoter and gene body of *MyoD*, but not at the core enhancer itself, suggesting that eRNA transcripts facilitate RNA PolII recruitment to the promoter of the target gene. In this study, we show that *Peblr20* recruits TET2 and facilitates eRNA transcription by inducing* Pou5F1* enhancer demethylation.

Transcriptional-regulatory circuitries may exhibit notable differences between human and mouse pluripotent stem cells. For example, human endogenous retrovirus subfamily H (HERVH) can produce lncRNAs that are highly expressed in human pluripotent cells. HERVH lncRNAs interact with Pou5F1 and coactivators in the regulatory network of pluripotency 5, 6. By genome browser alignment, it appears that *Peblr20* is conserved between mouse and rat, but not in human. Thus, *Peblr20* may function as a rodent-specific chromatin lncRNA to maintain the pluripotency identity. Whether there are pluripotency-associated lncRNAs that function using a similar mechanism specifically in human would need to be answered in the future.

**Figure 9:**
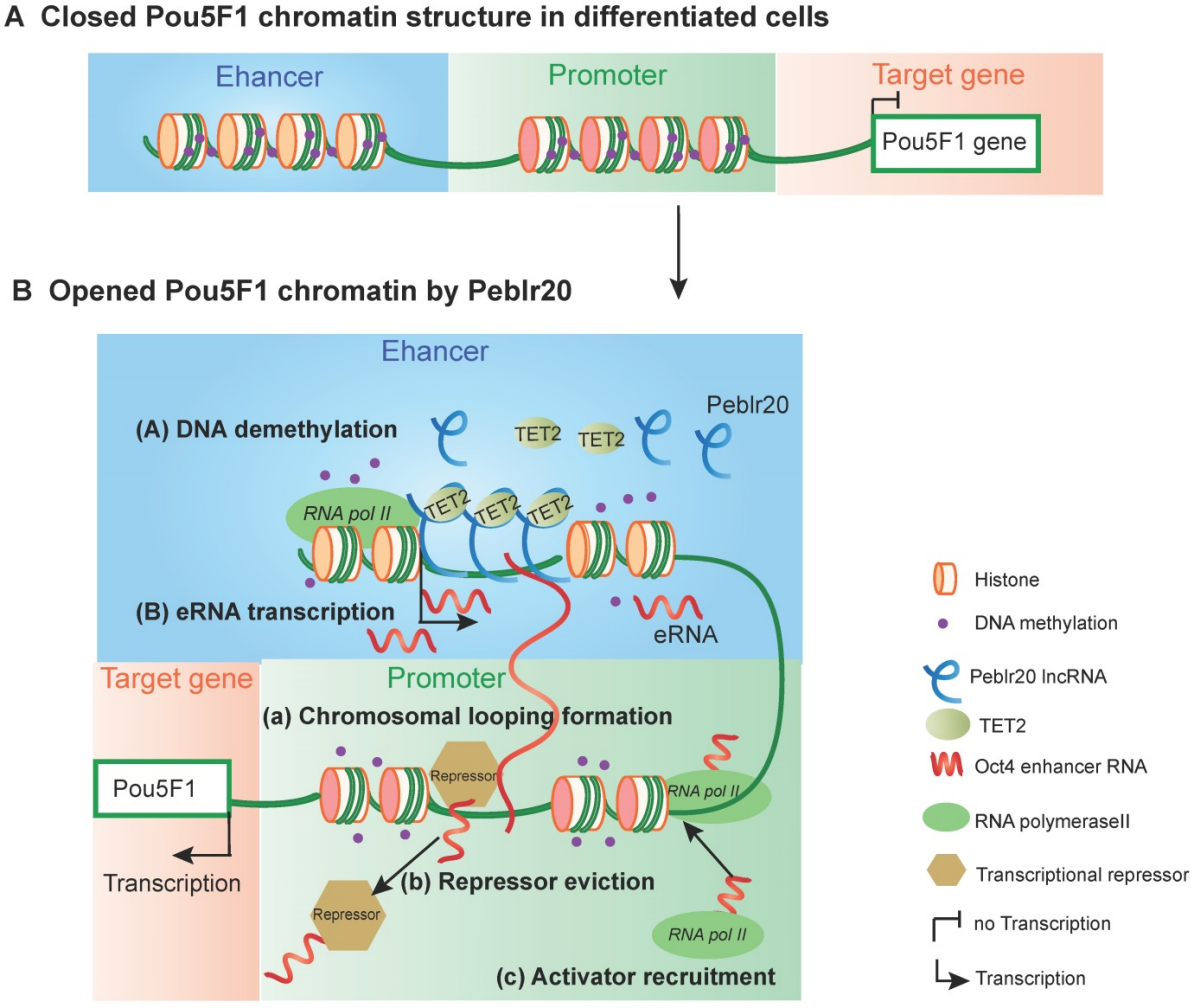
** The putative model of *Peblr20* in regulating pluripotency. (A)** Dormant *Pou5F1* gene locus in differentiated cells. The CpG islands of *Pou5F1* enhancer and promoter region are methylated leading to closed chromatin structure. **(B)** Active *Pou5F1* gene locus after *Peblr20* overexpression. Overexpressed *Peblr20* lncRNA recruits TET to *Pou5F1* enhancer loci, causes enhancer demethylation, leads to active enhancers which can transcribe functional enhancer RNAs. Subsequently, eRNAs activate the promoter by facilitating loop formation of enhancer and promoter, eviction of transcriptional repressors and recruitment of transcriptional activators. eRNA: enhancer RNA, RNA pol II: RNA polymerase II.

In conclusion, this study describes *Peblr20* as a critical pluripotent lncRNA that functions by epigenetically activating the enhancer RNA pathway (**Fig. 9**). *Peblr20* is specifically activated during the process of reprogramming. Once transcribed, *Peblr20* interacts in *trans* with regulatory elements of multiple stemness genes. *Peblr20* binds to the *Pou5F1* enhancers and recruits TET2 demethylase, leading to the activation of the enhancer RNA (eRNA) pathway. Thus, *Peblr20* utilizes a novel *trans* epigenetic eRNA mechanism to control the fate of stem cells.

### Data availability

The RNA-seq data generated in this study have been deposited in NIH GEO databases with accession number GSE116605, including 1). PSC RNA-seq.fq.gz (GSM3243668, iPSC RNA sequencing fastq data); 2). FIB RNA-seq.fq.gz (GSM3243669, Fibroblast RNA sequencing fastq data). The RAT-seq data generated in this study have been deposited in NIH GEO databases with accession number GSE101765, including 1). *Peblr20* RAT.fq.gz (*Peblr20*-specific primer RAT-seq library fastq data in iPSC); 2). PSC RAT Ct34.fq (GSM3244899, random control RAT-seq library fastq data in iPSC); 3). Palr35 RAT-seq.fq (GSM3244900, control lncRNA Palr35 RAT-seq library fastq data in iPSC). Palr35 was used as a control lncRNA as it is differentially expressed in reprogramming, but shows no interaction with core stem cell factor genes.
