# Supplementary material for: Genome-wide interaction target profiling reveals a novel Peblr20-eRNA activation pathway to control stem cell pluripotency
Source: Theranostics. 2020 Jan 1;10(1):353–70. doi: 10.7150/thno.39093 (PMC6929617; doi:10.7150/thno.39093)
Supplement: Supplementary file 1 — Supplementary figures and tables. [file thnov10p0353s1.pdf]

Genome-wide interaction target profiling reveals a novel *Peblr20*-eRNA activation pathway to control stem cell pluripotency

Cong Wang[1,2], Lin Jia[1,2], Yichen Wang[1,2], Zhonghua Du[1,2], Lei Zhou[1], Xue Wen[1], Hui Li[1], Shilin Zhang[1,2], Huiling Chen[2,3], Naifei Chen[1,2], Jingcheng Chen[1,2], Yanbo Zhu[1,2], Yuanyuan Nie[1], Ilkay Celic[2], Sujun Gao[1†], Songling Zhang[1†], Andrew R. Hoffman[2*†], Wei Li[1*†], Ji-Fan Hu[1,2*†], Jiuwei Cui[1†*]

[1] Stem Cell and Cancer Center, The First Hospital of Jilin University, Changchun, Jilin 130061, P.R. China

[2] Stanford University Medical School, VA Palo Alto Health Care System, Palo Alto, CA 94304, USA

[3] Department of Endocrinology, Xiangya Hospital, Central South University, Changsha, Hunan, P.R. China

Supplementary materials:

Figure S1. The genome-wide target analysis of Peblr20 by RAT-Seq.

A. Profiling pluripotent lncRNAs by integrating RNA-Seq and RAT-Seq. The conventional RNA-Seq approach identifies thousands of lncRNAs that are differentially expressed during pluripotent reprogramming. A modified RAT-seq approach was performed to map the genome wide interacting target genes for the selected lncRNA. It was hypothesized that as a pluripotent lncRNA, it should not only be activated in reprogramming, but also be able to blind to and thereby regulate multiple gene targets of core stem cell factors. Using this strategy, we identified Peblr20 as a pluripotent lncRNA, because it is activated in iPSCs and binds to multiple stemness gene targets.

B . Genetic features of Peblr20 RAT sequencing. More than 50% of Peblr20 binding sites were genomic intergenic regions and 1.78% was promoter elements.

C. Interaction of Peblr20 at the Pou5F1 locus. The RAT-seq data were uploaded onto UCSC genome browser by CYVERSE. Control: the RAT library was constructed with random oligo primers. Peblr20: the RAT library was constructed using Peblr20 complementary primers; 5'-Enh: 5'-enhancer; E1-E5: Pou5F1 exons 1-5; 3'-Enh: 3'-enhancer. Note the enriched binding of Peblr20 lncRNA at Pou5F1 3' enhancer, 5' enhancer, and exon5.

D. Interaction of Peblr20 at the Sox2 locus. Peblr20 binds to the Sox2 5'-enhancer. area.

E. Binding of Peblr20 to the CpG islands of the KLF4 gene.

Figure S2. Peblr20 lncRNA gene structure.

A. The 5’- and 3- RACE results. The Peblr20 5’- and 3’-ends were amplified by nested PCR, cloned into pJet vector, and sequenced. The 5’-end was different from 1700097N02Rik lncRNA (shown in red). GSP: gene specific primer.

B. Sequences of 1700097N02Rik and Peblr20. Peblr20 lncRNA is an alternative splicing variant of 1700097N02Rik, but with only two exons (746bp).

Figure S3. Differential expression of Peblr20 RAT-seq genes.

A. Activation of lncRNA Peblr20 in iPSCs.
B. Differential expression of Peblr20 RAT-seq target gene Pou5F1 between iPSCs and fibroblasts.
C. Differential expression of Peblr20 RAT-seq target gene Sox2 between iPSCs and fibroblasts.
D. Differential expression of Peblr20 RAT-seq target gene Tfcp2I1 between iPSCs and fibroblasts.
E. Differential expression of Peblr20 RAT-seq target gene Etl4 between iPSCs and fibroblasts.
F. Differential expression of Peblr20 RAT-seq target gene Tgm1 between iPSCs and fibroblasts.

Figure S4. Peblr20 overexpression and knockdown.

A. The Peblr20 shRNA plasmid. Peblr20 was knocked down by two Peblr20 shRNAs under the control of the H1 and U6 promoters, respectively, in a lentiviral pGreen vector.
B. Single colony selected after Peblr20 knocked down in iPSCs. After Peblr20 shRNA lentiviral transfection, iPSCs were selected by puromycin. Single colonies emitting green signal were picked up and collected for RT- Q-PCR.
C. The Peblr20 overexpression plasmid. Peblr20 was amplified by PCR according to 5'- and 3'-RACE and was cloned into lentiviral pCMV-DsRed/Puro vector. DsRed was used as the tracking marker.
D. Transfection of lenti pCMV-DsRed/Puro-Peblr20 in fibroblasts. fibroblasts were transfected by lenti DsRed-Peblr20 plasmid and selected by puromycin for 12 days. Note that nearly 90% of Fib were successfully transfected and emitted red signal.

Figure S5. Peblr20 induces DNA demethylation in the Pou5F1 promoter.

A. CpG sites in the Pou5F1 promoter. The status of DNA methylation at CpG3 site can be quantitated by restriction enzyme HpyCH4IV to separate the methylated and unmethylated DNAs. The restriction enzyme will not cut the unmethylated ACGT when the treatment of sodium bisulfite converts it into ATGT.
B. The methylation status of CpG3 (site 6). CpG3 was amplified by specific PCR primers, digested by HpyCH4IV, and quantitated by density scanning. Band A: unmethylated products that were not cut by the enzyme; Bands B and C: methylated products that were cut into two bands. Gray values (GV)

of these gel bands were measured by ImageJ and used for quantitation of methylation level(right). Methylation level=GV(B+C)/GV(A+B+C). Measurements were performed three times and statistically significant differences by Student's t test are indicated by *** indicating $P < 0.001$.

Table S1. Oligonucleotide primers

Table S1. Oligonucleotide primers used for PCR			
**ID**	**Oligo Name**	**Oligo Sequence**	**Product Size**
RT-PCR			
Peblr20	JH4838	CTATGGGGGCCAGAGCTTGA	104bp
	JH4839	ACTCCACAACCCCGCCAGCT	
β-Actin	J880	CAGGTCATCACCATTGGCAATGAG	135bp
	J881	CGGATGTCCACGTCACACTTCATGA	
Pou5F1	JH116	CAATGCCGTGAAGTTGGAGAAG	179bp
	JH117	GGCTGAACACCTTTCCAAAGAGA	
Sox2	JH118	GGTTACCTCTTCCTCCCACTCCAG	193bp
	JH119	TCACATGTGCGACAGGGGCAG	
Nanog	JH120	TCTCCTCCATTCTGAACCTGAGC	150bp
	JH121	TGCTGGGATACTCCACTGGTGCT	
U6	JH4061	GTGCTCGCTTCGGCAGCACATATA	103bp
	JH4062	ATATGGAACGCTTCACGAATTTGCG	
RAT			
Peblr20 specific primers	JH4839	ACTCCACAACCCCGCCAGCT	
	JH4841	GATGATTCTACCCAGGATGCT	
Random primer	JH5849	ATGGACTGATGATCTTATGC	
	JH5850	TACATAGTAGATCAGATACT	
Pou5F1 binding			
A	JH4348	CTGAGTCCTCTGCAAGATGC	137bp
	JH4349	CCAAGGCACCTGCCTAGGATT	
B	JH4350	CAGATGAGCCAACAGGTCTG	125bp
	JH4351	CAGCAACTTTGTCTGAAGTCC	
C	JH4352	AGTTGTCCCCAGGGGAGCCAT	140bp
	JH4353	AAGGGGCCTGGGAGGGACTG	
D	JH5932	GGTTGGAGCCCAACCTATAG	112bp
	JH5933	GAAGGCGGCTGCCAGACAAT	
E	JH5934	TGCTGCTGAATGTCAGCCCT	159bp
	JH5935	CACCATGGAGGGAACCAACT	
F	JH4407	GTGGAGCAGGCGAAACTTGC	123bp
	JH4408	ATTCCATCGGCAGCCTCAGC	

Sox2 binding			
A	JH5936	TGCATTTCCCTGTCGTCATCTG	131bp
	JH5937	ATGCTTCCTTCTGCCCTCTGA	
B	JH5938	AGTCTGCTCTTCCTCGGAATG	133bp
	JH5939	CTGCAGAGTCTCGGAGAATGC	
C	JH5054	GAAGCGGCCGTTCATCGACG	229bp
	JH5055	GTGCGCGTAGCTGTCCATGC	
3'- and 5'-RACE			
3' GSP	JH5292	GATGGGTCCAGTCCTGTATGA	
3' nested GSP	JH5293	ACCTGTGAATGTGCCATCCAC	
5' GSP	JH5291	GAGACCTGTCAATCCACGTC	
5' nested GSP	JH4839	ACTCCACAACCCCGCCAGCT	
Lenti RsRed-Peblr 20			
Peblr20 F	JH5416	TCGGGCGCCAGATATCGAGAGTTCAAAGGAAGTTGGGCGA	852bp
Peblr20 R	JH5417	AGAATCGAAGAATTCCAGCATGGAAGCAGTTTATTTAGC	
shRNA			
shRNA1		GCCGTTGAGAGTTCAAAGGAAGTTG	
shRNA2		CTGGCTTGCTTTGCTTTGCTAAATA	
shCT		GCAGCAACTGGACACGTGATCTTAA	
Enhancer RNA			
5' eRNA1	JH4662	CAGCCCCTAGCCTTGGACCT	133bp
	JH4663	ACACGTCCCCAGCCAGAGATG	
5' eRNA2	JH4350	CAGATGAGCCAACAGGTCTG	124bp
	JH4351	CAGCAACTTTGTCTGAAGTCC	
3' eRNA1	JH5932	GGTTGGAGCCCAACCTATAG	112bp
	JH5933	GAAGGCGGCTGCCAGACAAT	
3' eRNA2	JH5934	TGCTGCTGAATGTCAGCCCT	159bp
	JH5935	CACCATGGAGGGAACCAACT	
DNA methylation			
CpG1	JH4878	TTTAGGGAGGTTGAGAGTTTTGGGT	242bp

	JH4879	CATACTCACCTCCCAATTTCTATAC	
CpG2	JH5896	TATTTTAGGGAAGTTTAGGGTAGGT	190bp
	JH5897	TCCCCAACTCTCCACCTCTCCTCA	
CpG3	JH4880	GGATAGGTCGAGAGGGTGTAGTGTT	285bp
	JH4881	CACCCTCTAACCTTAACCTCTAAC	
CpG4	JH5992	GGGATAGTTGGGGTTGGAGTTTAAT	160bp
	JH5993	ATAAACCCACCAAAACTCCCA	
ChIP-qPCR			
5' distal enhancer	JH4350	CAGATGAGCCAACAGGTCTG	125bp
	JH4351	CAGCAACTTTGTCTGAAGTCC	
5'proximal enhancer	JH6095	GTTCAGGGTAGGCTCTCTGCA	160bp
	JH6096	TCCTCAAAGACAGAGCCTCAG	
3' enhancer	JH5932	GGTTGGAGCCCAACCTATAG	112bp
	JH5933	GAAGGCGGCTGCCAGACAAT	
RIP-qPCR			
Peblr20_A	JH5437	TCCTTCAACTCCGTCCCCCACA	140bp
	JH5291	GAGACCTGTCAATCCACGTC	
Peblr20_B	JH6097	GGCACTTCCCAGGTGGTAGC	120bp
	JH6098	GGCACTTCCCAGGTGGTAGC	
Peblr20_C	JH4840	GCTGGCTCCTGCAAGATGCT	119bp
	JH4841	GATGATTCTACCCAGGATGCTC	
Peblr20_D	JH5293	GCTGGCTCCTGCAAGATGCT	162bp
	JH5417	GATGATTCTACCCAGGATGCTC	
Positive control	forward	GGGAGATACCATGATCACGAAGGT	110bp
	reverse	CCACAAATTATGCAGTCGAGTTTCCC	

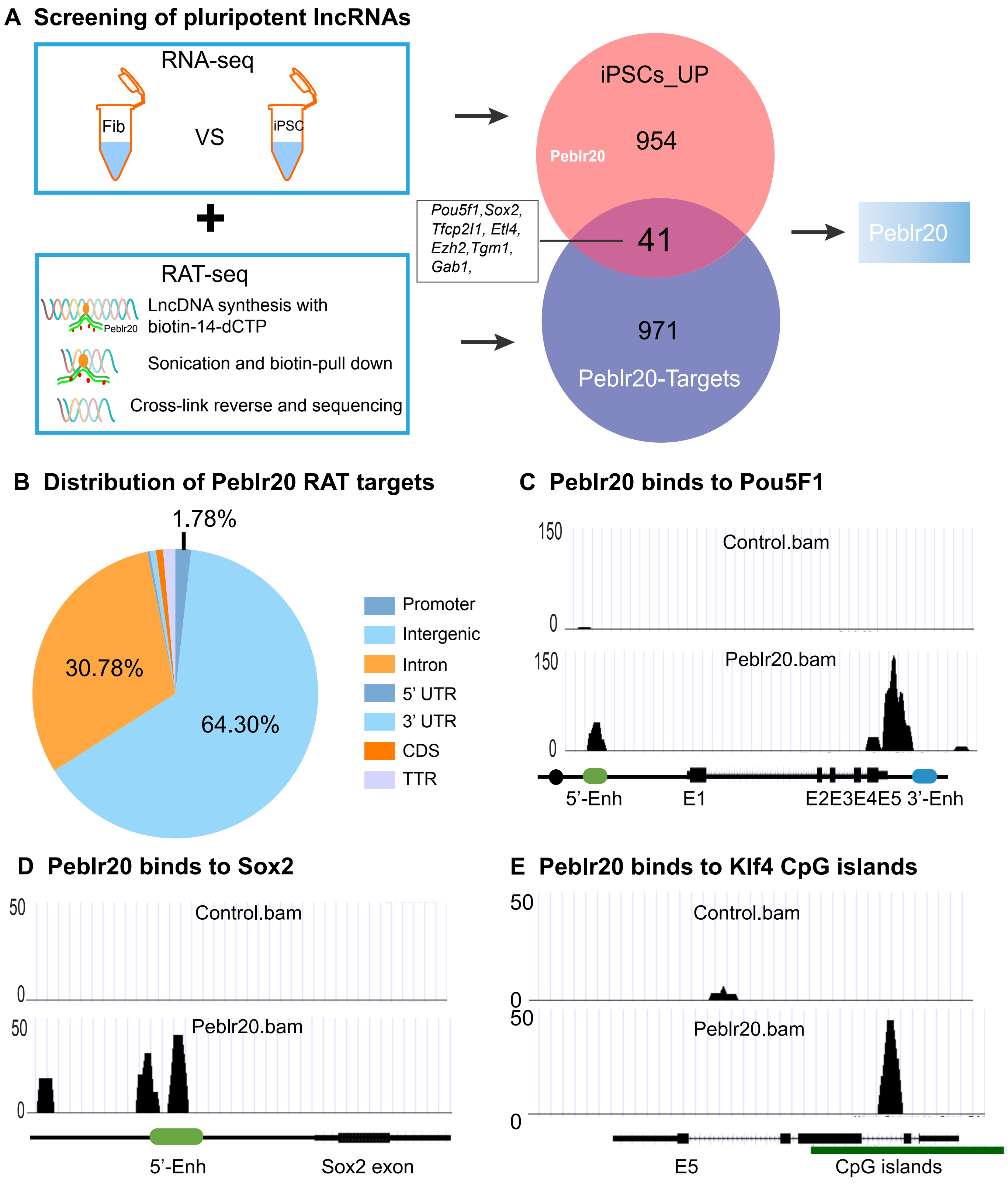

Figure S1. The genome-wide target analysis of Peblr20 by RAT-Seq

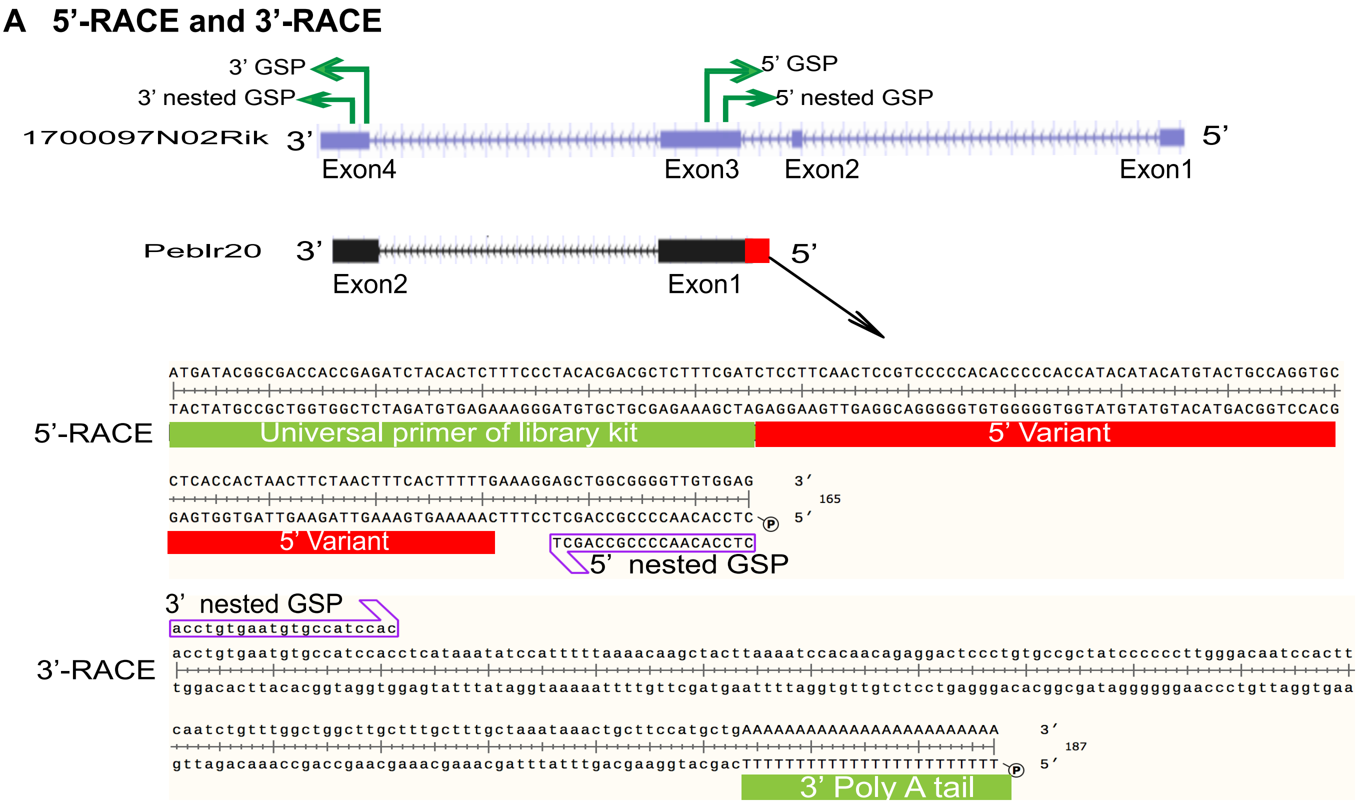

B Sequences of 1700097N02Rik and Peblr20

1700097N02Rik
gcccgcgggtgcctcgggtgcctcgggtgcctcgctatgggggccagagcttgaccatcatcagGcgctgggaacaccactctgggcctcagcaaaaccagcaagtactcttaaa
tgGagctggcggggttgtggagtaagatactcagacgtggattgacaggtctcaccagcacctgacaggatcccggcacttcccaggtggtagcttctacatccgccactccaagct
tcgcatccaccactcactcgcctgcctgtaaactggtgggaggctgtcttgaaccttagaccgccaaagcaggtcccaacagtggctgctctggtagagctgcccccagtctctgctgc
cactcggggcgcagccgttgagagtacaaaggaagttgggcggtggctcagccgttgagagttcaaaggaagttgggcgaggcgcctcacgcgctggctcctgcaagatgctgcg
cgcccgcgggtgcctcgggtgcctcgggtgcctcgctatgggggccagagcttgaccatcatcagGttttgctagagcatcctgggtagaatcatcttggaggatgggtccagtcctgt
atgatttgtaaaggaaagagtcaccttacctgtgaatgtgccatccacctcataaatatccattttaaaacaagctacttaaaatccacaacagaggactccctgtgccgctatcccccc
ttgggacaatccacttcaatctgtttggctggcttgctttgctttgctaaataaactgcttcca tgctgtctcttgacc

Peblr20
tccttcaactccgtcccccacacccccaccatacatacatgtactgccaggtgcctcaccactaacttctaactttcactttttgaaaggagctggcggggttgtggagtaagatactcaga
cgtggattgacaggtctcaccagcacctgacaggatcccggcacttcccaggtggtagcttctacatccgccactccaagcttcgcatccaccactcactcgcctgcctgtaaactggtg
ggaggctgtcttgaaccttagaccgccaaagcaggtcccaacagtggctgctctggtagagctgcccccagtctctgctgccactcggggcgcagccgttgagagtacaaaggaagt
tgggcggtggctcagccgttgagagttcaaaggaagttgggcgaggcgcctcacgcgctggctcctgcaagatgctgcgcgcccgcgggtgcctcgggtgcctcgggtgcctcgctat
gggggccagagcttgaccatcatcagGttttgctagagcatcctgggtagaatcatcttggaggatgggtccagtcctgtatgatttgtaaaggaaagagtcaccttacctgtgaatgtgc
catccacctcataaatatccattttaaaacaagctacttaaaatccacaacagaggactccctgtgccgctatccccccttgggacaatccacttcaatctgtttggctggcttgctttgctttg
ctaaataaactgcttccatgct

Figure S2. Peblr20 lncRNA gene structure

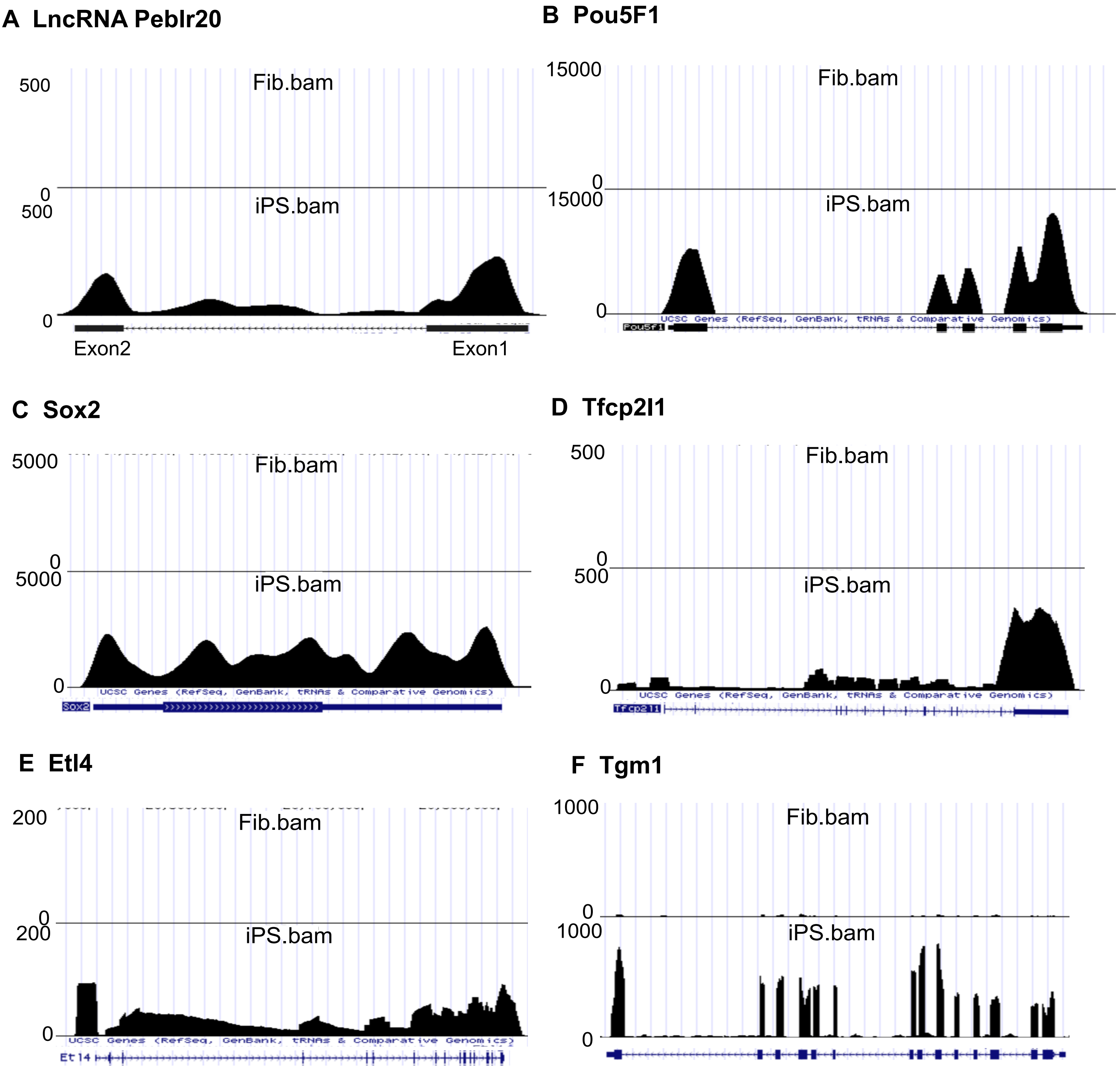

Figure S3. Differential expression of Peblr20 RAT-seq target genes

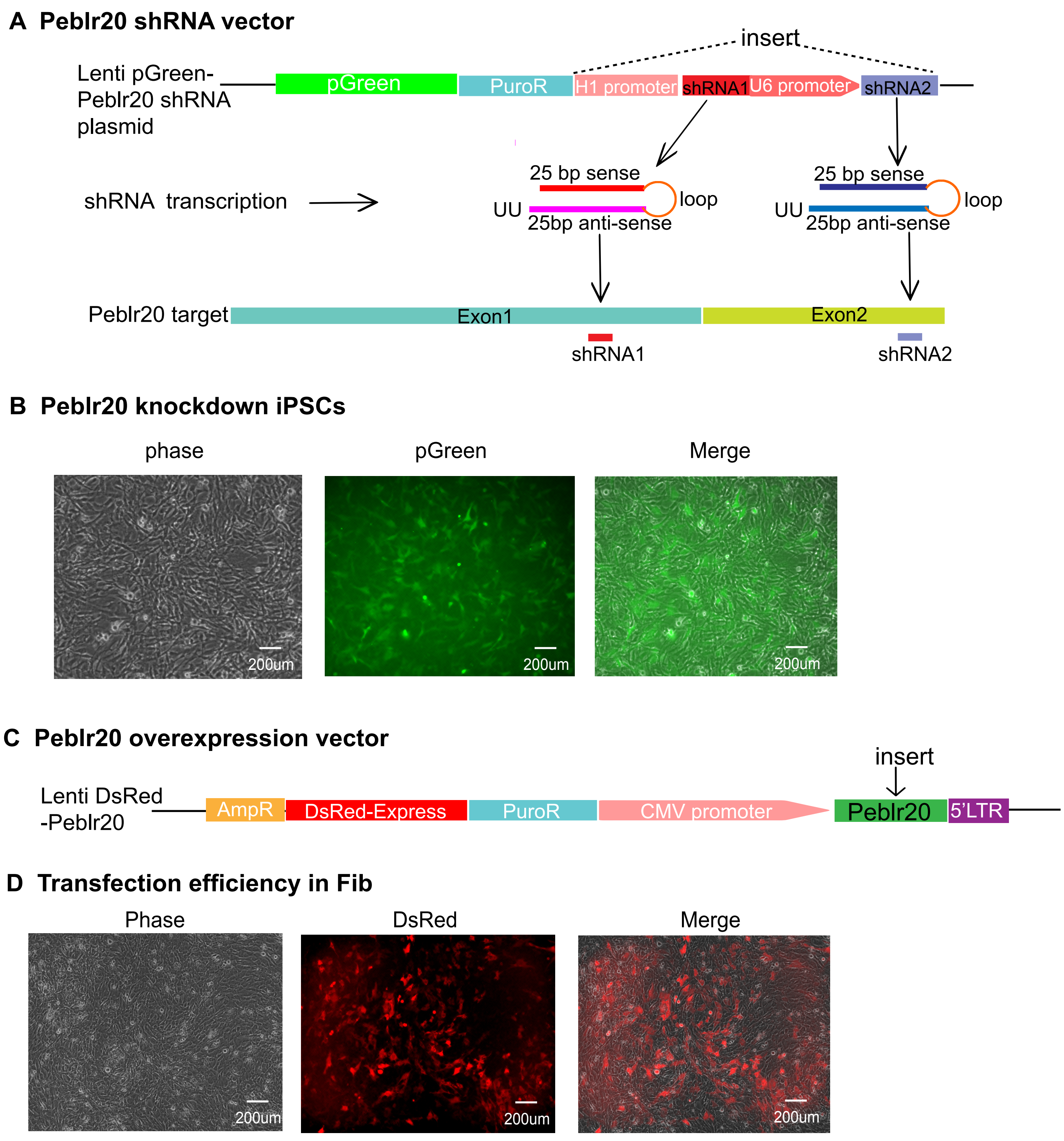

Figure S4. Peblr20 overexpression and knockdown

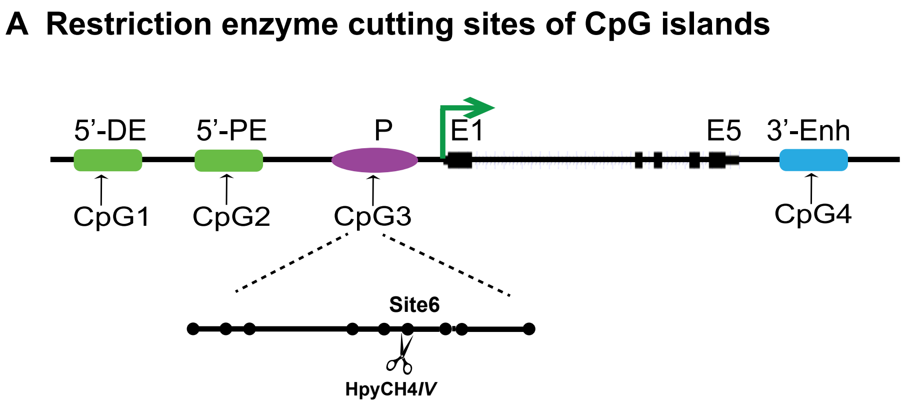

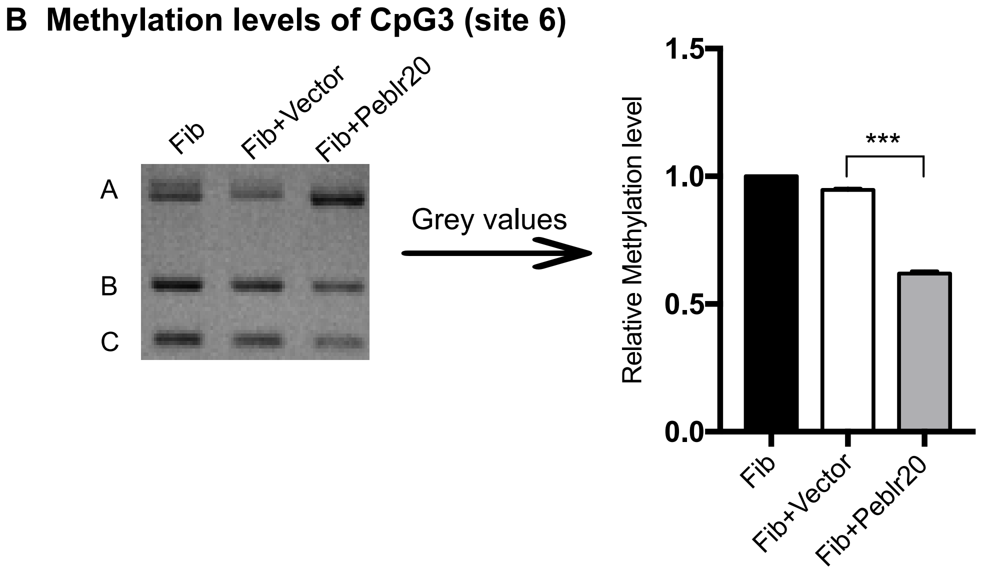

Figure S5. Peblr20 induces DNA demethylation in the Pou5F1 promoter
